# The primary pathway for lactate oxidation in *Desulfovibrio vulgaris*

**DOI:** 10.3389/fmicb.2015.00606

**Published:** 2015-06-26

**Authors:** Nicolas Vita, Odile Valette, Gaël Brasseur, Sabrina Lignon, Yann Denis, Mireille Ansaldi, Alain Dolla, Laetitia Pieulle

**Affiliations:** ^1^CNRS, LCB-UMR7283, Aix-Marseille UniversitéMarseille, France; ^2^CNRS, IMM-FR3479, Plate-forme Protéomique-IBISA Marseille-ProtéomiqueMarseille, France; ^3^IMM-FR3479, Plate-forme TranscriptomiqueMarseille, France; ^4^Laboratoire de Chimie Bactérienne, Institut de Microbiologie de la Méditerranée, Aix-Marseille UniversitéMarseille, France

**Keywords:** sulfate-reducing bacteria, *Desulfovibrio*, anaerobic lactate oxidation, lactate dehydrogenase, pyruvate-ferredoxin oxidoreductase

## Abstract

The ability to respire sulfate linked to lactate oxidation is a key metabolic signature of the *Desulfovibrio* genus. Lactate oxidation by these incomplete oxidizers generates reductants through lactate dehydrogenase (LDH) and pyruvate-ferredoxin oxidoreductase (PFOR), with the latter catalyzing pyruvate conversion into acetyl-CoA. Acetyl-CoA is the source of substrate-level phosphorylation through the production of ATP. Here, we show that these crucial steps are performed by enzymes encoded by a nonacistronic transcriptional unit named now as operon luo (for lactate utilization operon). Using a combination of genetic and biochemical techniques, we assigned a physiological role to the operon genes *DVU3027-28* and *DVU3032-33*. The growth of mutant Δ26-28 was highly disrupted on D-lactate, whereas the growth of mutant Δ32-33 was slower on L-lactate, which could be related to a decrease in the activity of D-lactate or L-lactate oxidase in the corresponding mutants. The *DVU3027-28* and *DVU3032-33* genes thus encode functional D-LDH and L-LDH enzymes, respectively. Scanning of the genome for lactate utilization revealed several lactate permease and dehydrogenase homologs. However, transcriptional compensation was not observed in any of the mutants except for lactate permease. Although there is a high degree of redundancy for lactate oxidase, it is not functionally efficient in LDH mutants. This result could be related to the identification of several operon enzymes, including LDHs, in the PFOR activity bands, suggesting the occurrence of a lactate-oxidizing supermolecular structure that can optimize the performance of lactate utilization in *Desulfovibrio* species.

## Introduction

Sulfate-reducing microorganisms are anaerobic prokaryotes that are widespread in natural habitats, such as marine and freshwater sediments and soil as well as in the gastrointestinal tracts of many animals, including humans. They efficiently link global sulfur and carbon cycles because they use sulfate as an electron acceptor for the anaerobic oxidation of inorganic or organic substrates (Muyzer and Stams, 2008). As a result of this metabolism, large amounts of sulfide, which is highly reactive, corrosive and toxic, are produced, and they accumulate in the natural habitats of the bacteria. For example, SRMs can cause serious economic problems for the oil industry because of their involvement in biocorrosion as well as oil souring and gas deposits because of hydrogen sulfide production (Hamilton, 2003; Dinh et al., 2004; Muyzer and Stams, 2008). SRMs, particularly *Desulfovibrio* species, present health implications because they can act as opportunistic pathogens associated with primary bacteremia and abdominal infections, such as abscesses and cholecystitis (Goldstein et al., 2003; Berry and Reinisch, 2013). However, SRMs may also be beneficial because they can remove sulfate, heavy metals, and radionuclides, such as chromium and uranium, from waste streams (Valls and de Lorenzo, 2002; Lloyd, 2003; Klonowska et al., 2008). Members of the genus *Desulfovibrio* are the most frequently studied representatives of SRMs and particularly *Dv*H, which appropriate genetic, biochemical, and genomic tools have been developed for.

Energy conservation by *Desulfovibrio* strains grown with lactate as a carbon and electron source has been extensively investigated because electron flow from lactate oxidation linked to sulfate reduction provides robust growth on this substrate. Moreover, in natural environments, SRMs most likely use fermentation products, lactate and/or H_2_ released by facultative anaerobes. *Desulfovibrio* strains are incomplete oxidizers of lactate, and they produce acetate in nearly stoichiometric quantities to that of the added substrate (Postgate, 1984). Therefore, approximately 95% of the lactate oxidized by *Desulfovibrio* strains is used for energy generation, and the remainder is used to produce cell material (Noguera et al., 1998). As a terminal electron acceptor, sulfate has unique properties that provide SRMs access to growth niches unavailable to other microorganisms. To reduce sulfate, the stable oxidized form of sulfur must be activated by an ATP sulfurylase, resulting in the formation of adenosine phosphosulfate (APS; Keller and Wall, 2011). This activation occurs at the expense of two ATP molecules. Energy conservation by coupling the reduction of sulfate to the oxidation of lactate is a complex mechanism because both the primary oxidation and terminal sulfate reduction reactions are cytoplasmic. Because lactate oxidation yields two ATPs by substrate-level phosphorylation, a net energetic benefit can only be obtained by electrogenic proton translocation, which is associated with the electron transport chain for the reduction of sulfate (Keller and Wall, 2011; Pereira et al., 2011; Price et al., 2014), or by hydrogen, formate, or CO cycling (Odom and Peck, 1981; Voordouw, 2002; da Silva et al., 2013). However, it remains unclear which of these mechanisms is more important for generating energy.

The *Desulfovibrio* pathway for lactate oxidation is through pyruvate to acetyl-CoA and then from acetyl-CoA to acetate via acetylphosphate, which has sufficient energy to generate ATP by substrate-level phosphorylation. A flux analysis of the central metabolic pathways in *Dv*H showed that approximately 84% of the lactate was partially oxidized into acetate via acetyl-CoA and indicated that this route was a main source of ATP production for this bacterium (Tang et al., 2007). In addition, the lower than expected carbon flow relative to that of acetate (84%, actual; >90%, expected) could be explained by an acetyl-CoA oxidization pathway (relative flux, ∼5%; Tang et al., 2007). The route of lactate oxidation requires a permease for the transport of lactate into the cell and four different enzymes to convert lactate into acetate. In bacteria, lactate can be oxidized into pyruvate *via* membrane-bound NAD-independent lactate dehydrogenase (iLDH) or soluble NAD-dependent lactate dehydrogenase (nLDH), (Garvie, 1980). More recently, two novel NAD-iLDHs have been described in *Shewanella oneidensis* (Pinchuk et al., 2009). Partial purification from various *Desulfovibrio* strains indicates that lactate is mainly converted into pyruvate via a membrane-bound NAD-iLDH capable of delivering electrons directly to the menaquinone pool in the membrane. However, this enzyme has not been well characterized. Depending on their stereoselectivity, two types of enzymes, L-lactate and D-lactate dehydrogenase, have been identified, and they differ in their respective sensitivity to oxygen. The L-LDH from *Desulfovibrio desulfuricans* HL21 has been shown to be extremely unstable when exposed to oxygen (Stams and Hansen, 1982), whereas the D-LDH from *D. vulgaris* Miyazaki was apparently much more stable (Ogata et al., 1981). An oxygen stable D-LDH was identified as a Zn^2+^ flavoprotein in the hyperthermophilic archaeal sulfate reducer *Archaeoglobus fulgidus* (Reed and Hartzell, 1999). The next step involved in the decarboxylation of pyruvate is a key reaction catalyzed by PFOR. The characterization of *Desulfovibrio africanus* (*Da*) PFOR showed that the enzyme contains one TPP molecule and three [4Fe-4S]^2+/1+^ clusters (Pieulle et al., 1995; Chabrière et al., 2001). In *Dv*H, PFOR is a homo-octomer, or more precisely, a tetramer in dimeric form of the related enzyme found in *Da* (Garczarek et al., 2007). Subsequently, the enzymatic actions of Pta and AckA result in the conversion of acetyl-CoA into CoA and acetate (Heidelberg et al., 2004). Two distinct forms of AckA were purified from *D. vulgaris* Miyazaki (Yu et al., 2001) whereas, to date, the characterization of *Desulfovibrio* Pta has not been undertaken. It has been proposed that *Dv*H and *D. desulfuricans* have an “organic acid oxidation region” in the genome containing all the genes for the route of lactate oxidation (Pereira et al., 2007; Wall et al., 2008).

To better characterize the crucial lactate oxidation pathway of *Desulfovibrio*, we first analyzed the genome region of *Dv*H containing the gene encoding PFOR, and we confirmed that this gene was the first of a polycistronic unit of nine genes, all of which are functionally related. In particular, we demonstrated that two LDHs that catalyzed the oxidation of either D-lactate or L-lactate in *Dv*H were encoded by this operon. Finally, preliminary data suggested that all of the proteins interacted together in a cytoplasmic supermolecular structure that oxidizes lactate efficiently and produces ATP by substrate-level phosphorylation.

## Materials and Methods

### Bacterial Strains and Growth Conditions

*Desulfovibrio vulgaris* Hildenborough (Postgate, 1984) was grown anaerobically at 33°C in lactate/sulfate medium C (Postgate, 1984). *Dv*H deletion mutant strains (Δ26-28 and Δ32-33) were cultured in the same conditions as the wild-type (WT) strain, and routine cultures of the WT and mutant strains were performed in the presence of kanamycin (50 μg/mL; to avoid any contamination) and thiamphenicol (Tm, 20 μg/mL), respectively; however, for growth analyses and enzymatic assays, the antibiotics were omitted. Growth was monitored by following the optical density at 600 nm with a spectrophotometer (CO8000 Cell Density Meter, Labgene Scientific Instruments, France). Cultures were inoculated at 1% (v/v) in 10 mL (Hungate tubes) with cells in the stationary growth phase (OD_600_∼0.9). For each growth assay, three biological replicates were performed.

### RNA Preparation and Reverse Transcription

RNAs were prepared from a *Dv*H culture (30 mL) in the exponential growth phase (OD_600_∼0.4). The cells were harvested and resuspended in 200 μl 10 mM Tris-HCl (pH 8.0) buffer. Total RNAs were isolated using the High Pure RNA kit from RocheLife Science (Roche Diagnostic, France) according to the manufacturer’s instructions and an extra Dnase I digestion step to reduce the amount of contaminating DNA. The RNA quality was assessed by agarose gel electrophoresis, and the absence of DNA contamination was confirmed by PCR. RNA was quantified spectrophotometrically at 260 nm (NanoDrop 1000; Thermo Fisher Scientific, USA). For cDNA synthesis, 5 μg total RNA and 3 μg random primers (Invitrogen, USA) were mixed, heated to 70°C for 3 min and placed on ice. The cDNA synthesis mix [50 mM Tris-HCl (pH 8.3), 40 mM KCl, 6 mM MgCl_2_, 10 mM DTT, 0.3 mM dNTPs] was then added. The reaction mix (30 μl) was incubated for 5 min at 25°C, and 300 units of Superscript II reverse transcriptase (Invitrogen, USA) were then added. The reaction mix was incubated for 5 min at 25°C, then for 1 h at 42°C, and finally for 15 min at 70°C for heat inactivation, and the volume was adjusted to 100 μl with ultrapure water. For all transcriptional experiments, RNAs were prepared from two independent biological replicate cultures.

### PCR Analyses

The appropriate primer pair (0.5 μM each, Supplementary Table S1) was added to cDNA in PCR buffer along with 100 μM dNTPs and 0.75 U GoTaq DNA polymerase (Promega). The reaction continued for 30 cycles at 96°C for 30 s, 55°C for 30 s, and 72°C for 1 min for 1 kb amplified in a TGradient thermocycler (Biometra, Switzerland). As controls, PCR was run under the same conditions with *Dv*H genomic DNA (positive control) and purified RNA (negative control). The PCR products were electrophoresed on either a 1 or 2% agarose gel according to the size of the PCR products.

### Quantitative Real-Time-PCR for Transcriptional Analyses

Quantitative real-time PCR (qRT-PCR) analyses were performed on a CFX96 Real-Time System (Bio-Rad). The reaction volume was 15 μL and the final concentration of each primer was 0.5 μM. The cycling parameters of the qRT-PCR were 98°C for 2 min, followed by 45 cycles of 98°C for 5 s and 60°C for 10 s and a final melting curve from 65°C to 95°C to determine the specificity of the amplification. To determine the amplification kinetics of each product, the fluorescence derived from the incorporation of EvaGreen into the double-stranded PCR products was measured at the end of each cycle using the SsoFast EvaGreen Supermix 2X Kit (Bio-Rad, France). The results were analyzed using Bio-Rad CFX Manager software, version 3.0 (Bio-Rad, France). The RNA16S gene (*Dv16SA*) was used as a reference for normalization. For each point a technical duplicate was performed. The amplification efficiencies for each primer pairs were comprised between 80 and 100%. All of the primer pairs used for qRT-PCR are reported in Supplementary Table S2.

### Determination of Transcriptional Start Sites

Total RNA extracted from the *Dv*H strain was hybridized with primer DVU3025rev, which is complementary to the DNA region located upstream of the ATG of *Dv*H (Supplementary Table S1). Primer DVU3025rev was ^32^P labeled using [γ^32^P-ATP] and T4 polynucleotide kinase (Biolabs, UK). In total, 5 μg RNA and 4 ng labeled primer were incubated along with 200 units of Superscript^TM^ III reverse transcriptase (Invitrogen, USA) for 50 min at 55°C and then 10 min at 70°C to inactivate the enzyme. The sequencing ladder was generated by direct sequencing of a PCR fragment obtained with the same labeled primer and a forward primer, DVU3025fwd. The sequencing reaction was performed using the Thermo Sequenase^TM^ Cycle Sequencing Kit (USB Corporation, USA). The extension and sequencing products were separated on a 6 M urea 8% acrylamide (19:1) gel.

### PFOR and LDH Activity Stains

For the in-gel activity assay, all of the steps were performed in a Jacomex anaerobic chamber. The crude extract of *Dv*H was prepared as follows. After reaching the mid-exponential phase, the cells were harvested by centrifugation (10,800 *g*, 20 min, 4°C) and the pellet was suspended with 50 mM Tris-HCl (pH 8.5) buffer. The cells were then passed through a French press cell at 1000 psi and centrifuged for 60 min at 20,000 *g* at 20°C. The crude extract was then separated through a non-denaturating 7% polyacrylamide gel at 20°C. PFOR activity was located by immersing the anaerobic gel in 50 mM Tris-HCl (pH 8.5) buffer containing 0.1 mM CoA, 20 mM pyruvate, 16 mM dithioerythritol, and 2 mM methyl viologen. After the blue band(s) of PFOR appeared, tetrazolium solution (2.5%, w/v) was added to preserve the electrophoresis pattern of PFOR in aerobiosis. For LDH activity, the anaerobic gel was incubated in 10 mM Tris-HCl (pH 8.5) buffer containing 65 μM phenazine methosulfate, 2 mM D-lactate, 2 mM L-lactate, 10 mM MgSO_4_, and 320 μM 3[4,5-dimethylthiazol-2,yl]-2,5,diphenyl tetrazolium (MTT). After 30 min, the reaction was stopped by the addition of HCl to a concentration of 0.1 M. LDH activity was detected by the appearance of blue bands, which formed as MTT was reduced to formazan, an insoluble compound (Reed and Hartzell, 1999). Activity bands were excised and incubated at room temperature for 15 min in Tris-HCl 75 mM (pH 8.8) buffer containing 6 M urea, 29.3% glycerol (v/v), 2% SDS (v/v), 1% bromophenol blue (w/v) and 1% dithiothreitol (w/v). After incubation, the proteins were separated by sodium dodecyl sulfate polyacrylamide gel electrophoresis (SDS-PAGE, 12%) and silver stained. Bands of interest were excised and then subjected to tryptic digestion and tandem mass spectrometry (MS/MS) analyses.

### In-Gel Trypsin Digestion of Proteins

Pieces of electrophoresis gel were transferred to a 96-well microplate (Greiner) for sample digestion. A robotic workstation (Freedom EVO 100, TECAN, Switzerland) was used to perform automated sample preparation, which including the following steps: washing, reduction, and alkylation, digestion by trypsin (proteomics grade, Sigma, USA), extraction and drying of mixed peptides (Maisonneuve et al., 2009).

### MS/MS Analyses

Digested peptides were analyzed by liquid chromatography (Ultimate NCS3500, Dionex, USA) coupled to an LCQ-DECA*^XP^* ion trap mass spectrometer (Thermo Fisher, USA) mounted with a nanospray ionization source (Thermo Finnigan, USA) as previously described (Maisonneuve et al., 2009). Protein identification was performed by TurboSEQUEST using the non-redundant National Center for Biotechnology Information [NCBI database restricted to *Desulfovibrio* (48,103 entries), and the identification was validated when at least two unique peptides of rank 1 (corresponding to a protein score ≥20)] were found. Mass spectrometry analyses were also performed on a LTQ Velos Orbitrap mass spectrometer (Thermo Fisher, USA) equipped with a nanospray ion source and coupled to a nanoflow Ultimate NCS 3500 (Dionex) high-performance liquid chromatography (HPLC) system. Tryptic peptides were dissolved in 2% acetonitrile/0.05% TFA in water, desalted on a C18 nanotrap and separated onto a C18 column (Acclaim PepMap RSLC, 75 μm × 150 mm, 2 μm, 100 Å, Dionex, USA) using a linear gradient from 4 to 55% of mobile phase B (20% water, 80% acetonitrile/0.1% formic acid) in A (0.1% formic acid in water) for 30 min. The peptides were analyzed in positive ion mode using one first-scan event full MS in the Orbitrap at 30,000 resolution, which was followed by one scan event of a collision induced by dissociation of the 10 top ion parents (MS/MS fragment analysis in the Orbitrap at 7,500 resolution). Processing of the spectra was performed with Proteome Discoverer software (Thermo Fisher Scientific), and a protein search was performed by MASCOT using the following parameters: NCBInr database 20120228 reduced to 3,669 *Dv*H sequences and variable modifications, which included carbamidomethylation (C); oxidation (M); ±8 ppm mass tolerance; ±0.8 Da fragment mass tolerance; and two missed cleavages. Proteins were identified when two unique peptides occurred with ion significance thresholds of *p* < 0.05.

### Construction of the Δ32-33 Deletion Mutant

The *cat* gene-containing fragment from pUC19Cm was amplified with the primers cat-f and cat-r, digested with *Xba*I and *Hind*III and ligated to similarly cleaved pNOT19 to yield pNOTCmΔ (Supplementary Table S1). The two ∼500 bp regions upstream and downstream of the *DVU3032* and *DVU3033* genes, respectively, were cloned into the pNOTCmΔ, which produced pNOTCmΔ32-33, and the mutagenic plasmid was transferred into *Dv*H by electrotransformation. Briefly, cells grown in 80 mL medium C (OD_600nm_ = 0.6–0.7) were pelleted by centrifugation, washed twice with sterile chilled anaerobic water, and resuspended in 250 μL chilled anaerobic water. Fifty microliters of cell suspension was electroporated (1900 V; 250 Ω; and 25 μF; ECM 630 Electroporation system, BTX, Gentronix, San Jose, CA, USA) with 500 ng pNOTCmΔ32-33. After pulsing, the cell/DNA mixture was transferred to a serum bottle with 30 mL medium C and then incubated at 33°C. Tm was added after 6 h, and the serum bottle was further incubated at 33°C for 5–6 days. Cells were then plated on PE-agar (Postgate, 1984) petri dishes containing Tm and then incubated in an anaerobic chamber until colonies appeared. The colonies were screened by PCR to determine whether the appropriate deletion strain was obtained. The desired mutants, which required a double recombinational event, were predominantly obtained with this protocol.

### Assay of LDH Activity

For analysis of LDH activity in living bacteria, *Dv*H was grown in medium C to the late log phase (OD_600nm_∼0.7–0.9) and washed four times in buffer 50 mM Tris-HCl (pH 8.5) to eliminate excess sulfide. All of the steps were performed using an anaerobic chamber. The cells were resuspended in the same buffer containing 10% Triton X-100 to increase the accessibility of the reactives. Suspensions were then incubated for 20 min at 37°C. LDH was assayed at 30°C by a 2,6-dichlorophenolindophenol (DCPIP) linked assay as previously described (Thomas et al., 2011). Oxidation of lactate (sodium salts of D-lactate and/or L-lactate at 10 mM) was detected anaerobically by the reduction of DCPIP. Detergent-treated bacteria were assayed in a 1 mL reaction mixture containing 50 mM Tris-HCl (pH 8.5), 0.5% Triton X-100, and 70 μM DCPIP. The change in OD_600nm_ was measured with a Beckman DU40 spectrophotometer in an anaerobic sample cuvette. LDH activity was expressed in nanomoles of lactate oxidized per minute and per milligram of protein and based on an extinction coefficient for DCPIP of 21 mM^-1^cm^-1^.

## Quantification of Lactate for Evaluation of the Substrate Uptake Rate

Cells were grown in medium C with D-lactate or/and L-lactate to the late log phase, and 1 mL of culture was sampled and centrifuged for 15 min at 10,000 *g*. Extracellular lactate was quantified in supernatant by HPLC using a SpectraSERIES P100 pump equipped with a SpectraSystem RI-150 detector and an Aminex HPX-87 column C18 (Bio-Rad, France). The column temperature was 37°C, and eluent (H_2_SO_4_, 0.005 N) was used at a flow rate of 0.6 mL min^-1^.

## Results

### Predicted Function and Structure of the Organic Acid Oxidation Region Genes

**FIGURE 1:**
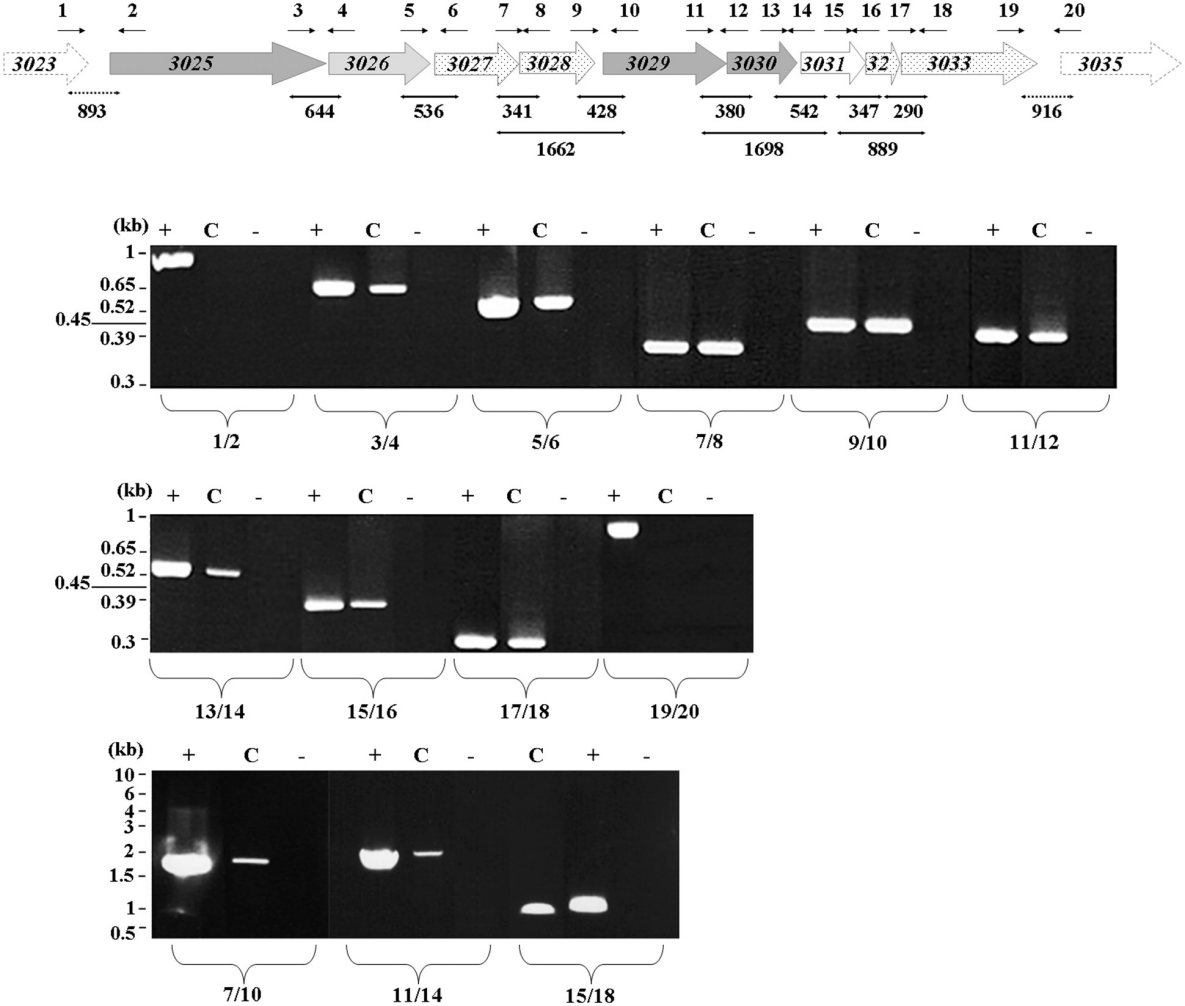
**RT-PCR analysis of the *Dv*H “organic acid oxidation region” genes.** All of the ORFs described in the microbesonline database (http://www.microbesonline.org/) are represented except for the two ORFs *DVU3024* and *DVU3034*, which encode putative small unconserved proteins (55 and 33 amino acids, respectively) and do not display the Shine–Dalgarno (SD) sequence upstream of the start codon. For *DVU3028*, another start codon was observed 27 nucleotides upstream of the previously reported (Heidelberg et al., 2004) and proposed codon (Price et al., 2011). Products were analyzed by electrophoresis on agarose gel and obtained from cDNAs PCR amplified by the addition of primer pairs (Supplementary Table S1) to reveal transcriptional links between successive genes. (–) PCRs performed on RNAs; (+) PCRs performed on genomic DNA; and (C) PCRs performed on cDNAs. Numbered arrows indicate the positions of the primers used. Thick black arrows below the gene map indicate the transcriptional links revealed by RT-PCR analysis, and dotted arrows indicate the absence of links. The expected size of the PCR fragments is reported in base pairs.

**FIGURE 2:**
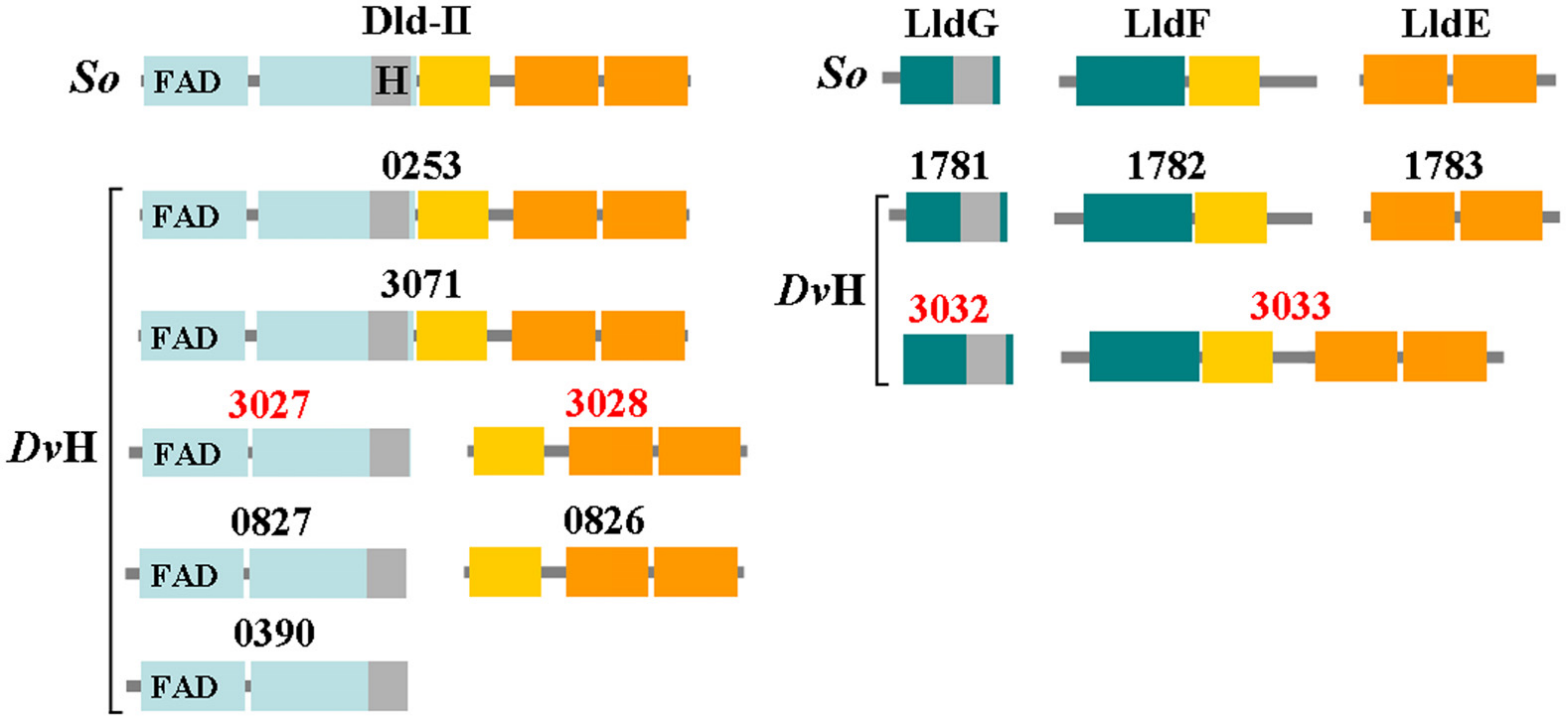
**Domains and motifs found in the Dld-II and LldEFG orthologs in the *Dv*H genome (according to the Pfam database).** The FAD-binding and FAD-linked oxidase domains (Pfam accession nos. PFO1565 and PFO2913, respectively; blue). H highlighted in gray corresponds to the essential histidine conserved in enzymes that binds to lactate (Griffin et al., 1992). The 4Fe-4S dicluster domain (PF13183; yellow). Two CCG domains (PFO2754; orange). Two DUF162 domains (PFO2589) were identified in all of the LldEFG orthologs (green). *So, Shewanella oneidensis*.

A schematic representation of the *Dv*H organic acid oxidation region consisting of nine open reading frames (ORFs) is represented in **Figure 1**, and the corresponding annotations^1^ are reported in **Table 1**. Certain ORFs are annotated as the encoding enzymes that are most likely involved in the phosphoroclastic reaction, including *DVU3025* (also called *por*), *DVU3029* and *DVU3030*, which encode PFOR, Pta and Ack, respectively. A sequence analysis of the Pta (DVU3029) revealed the presence of three conserved domains: a catalytic PTA_PTB protein domain, which is found in all Pta, and AAA and DRTGG domains, which are only found in class II enzymes (Campos-Bermudez et al., 2010). *DVU3026* encoded a putative lactate permease; however, annotation of the remaining ORFs was unclear: *DVU3031* encoded a conserved hypothetical protein consisting of the AAA and DRTGG domains but without a PTA_PTB protein domain, thus excluding putative Pta activity for this protein. DVU3027 and 3028 were annotated as a glycolate oxidase subunit and iron-sulfur cluster-binding protein encoding gene, respectively. However, their amino acid sequences suggested that they corresponded to two subunits of a flavin- and iron sulfur-containing oxidoreductase homolog of the monomeric D-iLDH (Dld-II), which is characterized in *Shewanella oneidensis* (Pinchuk et al., 2009) as already proposed in Pereira et al. (2011). Despite the low pairwise sequence identity (17% sequence identity, Supplementary Figure S1), DVU3027-28 consisted of the same protein domains and motifs, including the FAD-binding domain (Pfam accession no. PF01565), FAD-linked oxidase domain (PF02913), the 4Fe-4S dicluster domain (PF13183) and CCG domain (PF02754), (**Figure 2**). The C-terminal FAD-linked oxidase domain of DVU3027 contained a sequence close to the motif GEHGD and an essential histidine conserved in enzymes that bind lactate (Griffin et al., 1992). DVU3032 and DVU3033 were annotated as a conserved hypothetical protein and iron sulfur cluster-binding protein, respectively. However, their amino-acid sequences shared 26% amino acid sequence identity with the three subunits of the non-flavin iron-sulfur containing oxidoreductase (LldEFG, Supplementary Figure S2) of *S. oneidensis* (Pinchuk et al., 2009). Moreover, both proteins shared the same multi-domain composition, which is shown in **Figure 2**. In addition to the iron sulfur-containing domains, DVU3033 contained an N-terminal domain of unknown function (DUF162), and this protein domain was also detected in DVU3032. Therefore, this analysis suggests that *DVU3027-3028-3032-3033* genes are candidate genes for lactate utilization enzymes belonging to the *Shewanella* LDHs family, although with a different subunit organization resulting from either genes fusion or splitting.

**Table 1:** Annotation and proposed function of the “organic acid oxidation region” genes.

Locus tag	Annotation^1^	Proposed function	Gene name
DVU3025	Pyruvate-ferredoxin oxidoreductase	Pyruvate-ferredoxin oxidoreductase	*por*
DVU3026	L-lactate permease family protein	D,L-lactate permease	
DVU3027	Glycolate oxidase, subunit GlcD	D-lactate dehydrogenase subunit	*dldII-A*
DVU3028	Iron-sulfur cluster-binding protein	D-lactate dehydrogenase subunit	*dldII-B*
DVU3029	Phosphate acetyltransferase (pta)	Phosphate acetyltransferase	*pta*
DVU3030	Acetate kinase (ack)	Acetate kinase	*ack*
DVU3031	Conserved hypothetical protein	Unknown	
DVU3032	Conserved hypothetical protein	L-lactate dehydrogenase subunit	*lldG*
DVU3033	Iron-sulfur cluster-binding protein	L-lactate dehydrogenase subunit	*lldH*

*^1^Microbesonline database annotation (http://www.microbesonline.org/).*

**FIGURE 3:**
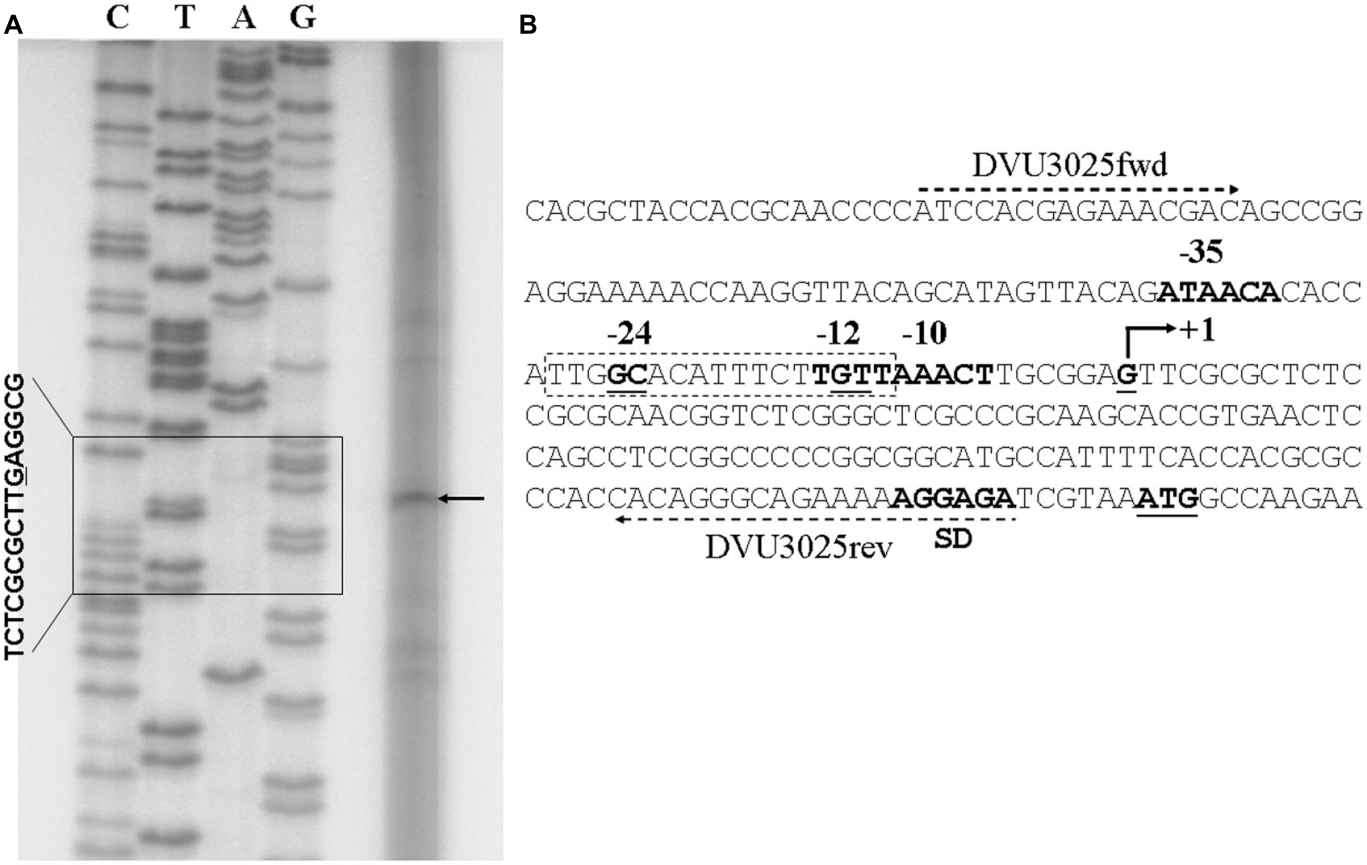
**Identification of signal transcription of the *Dv*H operon luo. (A)** Primer extension analysis. The arrowhead indicates the primer extension product generated using the primer DVU3025rev. Lanes C, T, A, and G correspond to the sequences read using the same primer. Sequence near the transcriptional +1 position is indicated (left). **(B)** Sequence of the upstream region of the *por* gene with the position of the +1 transcriptional start site; -10 and -35 boxes of a bacterial σ70 promoter were predicted by Softberry-BPROM (scores for the -10 and -35 boxes are 76 and 13, respectively). A σ54-regulated promoter was also predicted by PromScan (score 85). The sequence of this promoter is boxed, and it overlaps with the -10 box of the σ70 promoter. The predicted SD consensus and initiation codon for the *por* gene are shown in boldface and underlined, respectively. The forward and reverse primers used for transcription start site determination and sequencing of the region are indicated by dotted arrows.

The operon structure of the organic acid oxidation region was assessed by RT-PCR experiments on total RNA isolated from *Dv*H grown on lactate-sulfate medium. Upon retrotranscription of the operon transcript, amplification of the intergenic sequences demonstrated that the nine genes (from *DVU3025* to *DVU3033*) belonged to the same transcriptional unit, which is hereafter referred to the operon luo (for lactate utilization operon; **Figure 1**). The transcription start site of this operon was then mapped by primer extension assay. A single extension product was obtained, which located the transcriptional start point (nt 3,143,640) 12 bp downstream of the -12 element of the predicted σ54-dependent promoter and 7 bp downstream of the -10 element of the predicted σ70-dependent promoter (**Figure 3**).

### Two Dimeric LDHs are Encoded by the Operon Luo in *Dv*H

**FIGURE 4:**
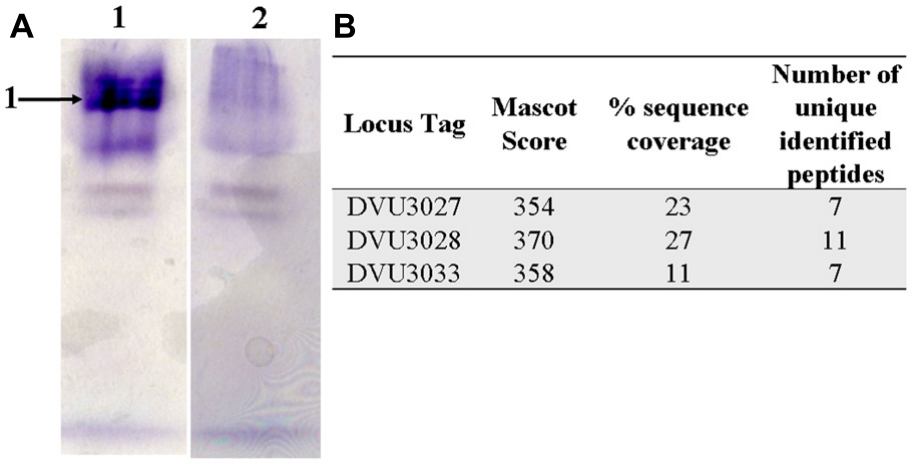
**In-gel lactate dehydrogenase activity and protein identification. (A)** Separation of *Dv*H proteins from crude extract on non-denaturing 7% PAGE and lactate dehydrogenase activity staining in the presence of D-lactate + L-lactate (lane 1) and in their absence (lane 2). **(B)** Identification by mass spectrometry of proteins in band 1 (A) after electrophoresis under denaturing conditions.

In-gel lactate dehydrogenase activity assays were performed on the crude extract of *Dv*H to confirm the prediction of DVU3027-3028 and DVU3032-3033 as lactate dehydrogenases. According to the oxygen sensitivity of *Desulfovibrio* LDH (Stams and Hansen, 1982), all of the steps were performed in an anaerobic chamber. After electrophoresis, incubation of the polyacrylamide gel with lactate showed an intense band near the top of the gel (**Figure 4A**). In the absence of any added lactate, only fuzzy bands were observed, which could be explained by the anaerobic conditions of the experiments; thus, the enzymes could have been maintained in a reduced state, which would have allowed for the reduction of the artificial acceptor without any exogenous substrate addition. The activity band was excised and electrophoresed on a polyacrylamide gel under denaturing conditions to identify the proteins by MS (data not shown), and DVU3027, DVU3028, and DVU3033 were identified among the more intense protein bands (**Figure 4B**) in a region of the gel that corresponded to the expected molecular weight of these proteins. It should be noted that DVU3032 was not detected. However, its 209 amino acid sequence, which contains 22 putative trypsin cleavage sites, generates an unsuitable peptide profile for reliable identification by MS.

Our analysis allowed us to specify the annotation of the ORFs constituting the operon luo and indicated that this operon encoded two heterodimeric LDHs; therefore, it could unite all of the components necessary for ATP production from lactate oxidation.

### Gene Deletion Mutagenesis Emphasizes the Stereospecificity and Predominant Role of Operon LDHs

To confirm the role of the inferred lactate utilization genes in *Dv*H, the respective chromosomal deletion mutants were tested for their ability to grow on lactate. Moreover, because *Dv*H was able to grow on either D-lactate or L-lactate, the ability of the mutants to grow with only one lactate stereoisomer was also tested. As reported in **Table 2**, all of the growth parameters were similar when WT cells were grown on a mixture of D-lactate + L-lactate or on only one stereoisomer. These results showed that *Dv*H could use D-lactate or L-lactate equally with the same efficiency.

**Table 2:** Growth parameters for the wild type (WT) and LDH mutants of *Dv*H.

Strain	Condition	Doubling time^1^ (Tg) (h)	Substrate uptake rate, q_s_^2^ (mol h^-1^ g^-1^)
WT	D- + L-lactate	4.99 ± 0.41	16.7 × 10**^-^**^3^
	D-lactate	5.38 ± 0.35	15.2 × 10**^-^**^3^
	L-lactate	5.20 ± 0.40	18.6 × 10**^-^**^3^
Δ26-28	D- + L- lactate	3.85 ± 0.38	19.5 × 10**^-^**^3^
	Dlactate	9.05 ± 0.10	6.8 × 10**^-^**^3^
	L-lactate	5.11 ± 0.33	21.0 × 10**^-^**^3^
Δ32-33	D- + L- lactate	5.80 ± 0.27	13.0 × 10**^-^**^3^
	D-lactate	5.10 ± 0.12	17.2 × 10**^-^**^3^
	L-lactate	15.16 ± 0.27	6.4 × 10**^-^**^3^

*^1^Doubling time values are calculated from three biological replicates.*

*^2^q_s_ = μ/Y, where μ (h**^-^**^1^) is the specific growth rate and Y (g/mol) is the molar growth yield ([synthetized biomass]/[consumed lactate]).*

**FIGURE 5:**
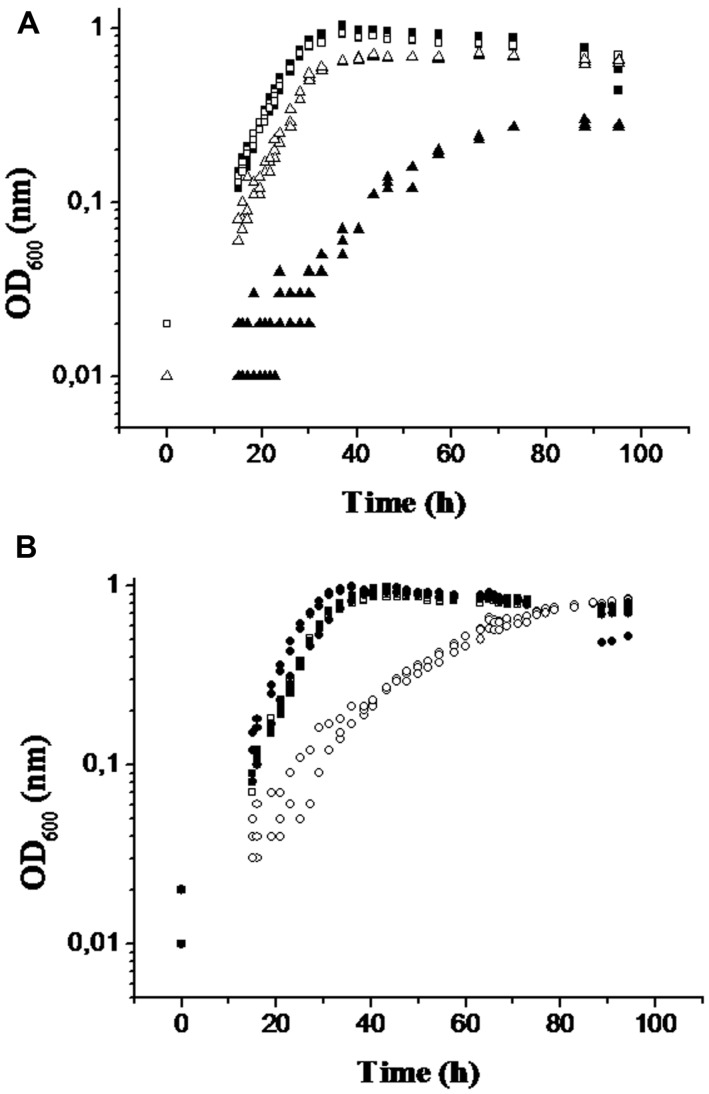
**Growth of the wild-type (WT) and mutant strains in the presence of the either D- or L-lactate stereoisomer. (A)** Cell growth of the WT, (square) and mutant Δ26-28 (triangle) in medium C containing D-lactate (black symbol) or L-lactate (white symbol). **(B)** WT (square) and mutant Δ32-33 (circle) in D-lactate (black symbol) or L-lactate (white symbol) PC medium. All of the conditions were performed triplicate, and the three corresponding curves are shown.

The growth of a mutant strain (Δ26-28; Prof. G. Voordouw, personal gift) that had the *DVU3027* and *DVU3028* genes deleted was analyzed. It should be noted that this deletion mutant strain also had *DVU3026* deleted. The deletion of these genes had no significant effects on the expression of the genes downstream from *DVU3029* to *DVU3033*, which was verified by qRT-PCR (data not shown). On D-lactate, the growth of the mutant Δ26-28 was critically impaired, with ∼70% less biomass (based on final OD_600nm_) accumulated and a twofold longer doubling time compared with its growth on D-lactate + L-lactate or L-lactate alone (**Figure 5A** and **Table 2**). Moreover, the substrate uptake rate for L-lactate was the same as that for the WT, whereas the uptake rate for D-lactate was much lower than that of the WT (2.2 times lower; **Table 2**). These results indicate that the *DVU3027* and *DVU3028* genes encoded an LDH specific for D-lactate. An analysis of the Δ32-33 mutant (*DVU3032* and *DVU3033* genes deleted) showed that its growth was only affected on L-lactate compared with that of the WT (**Figure 5B**). This growth disruption was mainly indicated by a doubling time that was longer for the mutant (∼15 h) than for the WT strain (∼5 h), and the same doubling time was obtained when the two strains grew on D-lactate (**Table 2**). In addition, the substrate uptake rate for L-lactate was 2.9 times slower for the mutant than for the WT strain, whereas it was quite similar for the two strains on D-lactate (**Table 2**).

To extend the genetic findings and provide biochemical evidence for the proposed stereospecificity, LDH activities were assayed in detergent-treated bacteria. The LDH activities measured in the Δ26-28 cells were similar to those in the WT cells when L-lactate was used as a substrate. However, with D-lactate as the substrate, the activity was decreased by approximately 75% in the mutant cells compared with that of the WT cells (**Table 3**). A decrease of 55% of the L-LDH activity was observed in the Δ32-33 mutant strain, and a decrease in D-LDH activity (∼ 38%) was also observed, although it was not correlated with the growth results because this deletion mutant grew at similar rates to that of the WT strain with D-lactate, whereas growth on L-lactate was greatly impaired.

**Table 3:** D- and L-LDH enzymatic activities in WT and LDH mutant cells of *Dv*H.

		LDH activity (nmol min^-1^ mg protein^-1^)		
Strain	Substrate	D- + L-lactate	D-lactate	L-lactate
WT		14.2 ± 2.4	13.5 ± 3.0	8.5 ± 2.9
Δ26-28		9.8 ± 1.8	3.3 ± 0.4	8.0 ± 2.2
Δ32-33		11.8 ± 3.5	7.2 ± 2.2	3.8 ± 1.0

All together, these data strongly suggest that DVU3027 and DVU3028 constitute a dimeric LDH specific for D-lactate and indicate that DVU3032 and DVU3033 are the two subunits of a LDH specific for L-lactate.

### Redundancy of Lactate Utilization Machinery in *Dv*H

Notably, the two deletion mutants only partially lost their ability to grow on one lactate stereoisomer alone, and significant oxidation of this stereoisomer was also measured in mutant cells. Thus, several LDH orthologs were proposed in the *Dv*H genome (Keller and Wall, 2011; Meyer et al., 2012). A sequence analysis allowed us to classify these orthologs into several groups (**Table 4**). The first group included DVU0826-27, DVU3071, DVU0390, and DVU0253, which are paralogs of DVU3027-28 in the Dld-II family. The second group included DVU1781-82-83, which is a paralog of DVU3032-33 in the LldEFG family. A schematic representation of these Dld-II and LldEFG orthologs based on the predicted Pfam domains showed that the multidomain organization was the equivalent for all of the homologs except for the protein DVU0390, whereas the predicted oligomeric nature of the enzymes was not equivalent (**Figure 2**). All of the sequences (except for the DVU0390 sequence) contained the structural features for FAD (in the case of Dld-II orthologs), [Fe-S] clusters and lactate binding consensus sequences required for lactate oxidation (**Figure 2**). An ortholog of L-lactate dehydrogenase from *Escherichia coli*, DVU2784, was identified, and it exhibited 32% sequence identity. This protein contained the HGGR motif for FMN binding and essential residue for enzymatic catalysis (Dong et al., 1993). In addition, DVU0600 and DVU1412 were found to exhibit approximately 40 and 50% sequence identity with fermentative LDHs from *Clostridium cellulolyticum* (Li et al., 2012) and *S. oneidensis* (Pinchuk et al., 2009), respectively.

**Table 4:** Redundancy of lactate oxidation enzymes in *Dv*H.

Lactate dehydrogenases	Putative name	Locus tag(s) of genes	Gene product annotation (http://www.microbesonline.org)
**D-lactate dehydrogenase**
		DVU3027-28	Glycolate oxidase, subunit GlcD (3027) Iron-sulfur cluster-binding protein (3028)
		DVU0826-27	Glycolate oxidase, iron-sulfur subunit, putative (0826) Glycolate oxidase, subunit GlcD, putative (0827)
		DVU3071	Oxidoreductase, FAD/iron-sulfur cluster-binding domain protein
		DVU0390	Glycolate oxidase, subunit GlcD, putative
	Dld-II	DVU0253	Oxidoreductase, FAD/iron-sulfur cluster-binding domain protein
**L-lactate dehydrogenase**
		DVU3032-33	Conserved hypothetical protein (3032) Iron-sulfur cluster-binding protein (3033)
	LldEFG	DVU1781-83	Conserved hypothetical protein (1781) Iron-sulfur cluster-binding protein (1782) Cysteine-rich domain protein (1783)
	L-LdD	DVU2784	Dehydrogenase, FMN-dependent family
**Fermentative LDHs**
		DVU0600	L-lactate dehydrogenase
	D-LdhA	DVU1412	D-isomer specific 2-hydroxyacid dehydrogenase family protein

**FIGURE 6:**
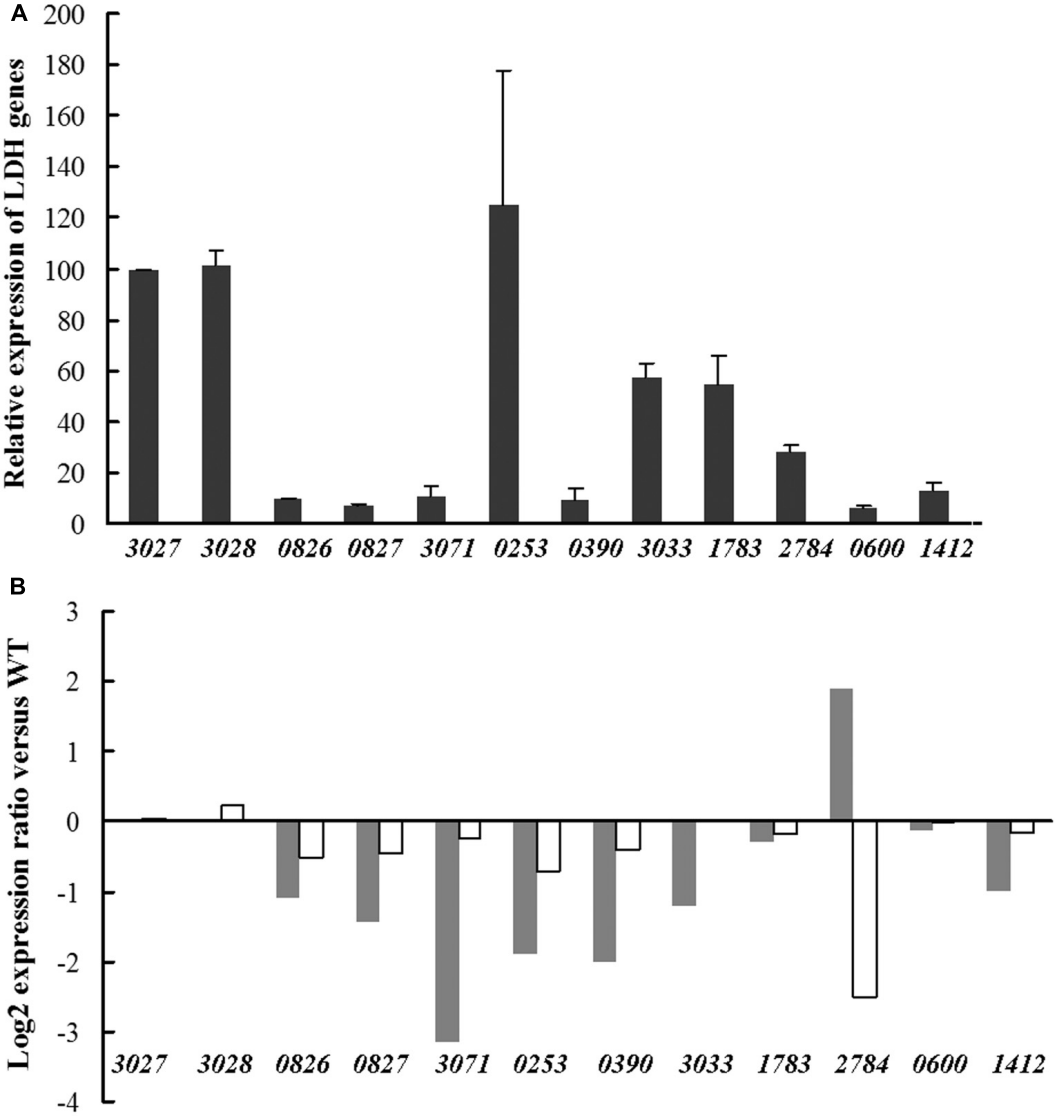
**Transcriptional analyses of predicted LDH encoding genes in *Dv*H. (A)** Relative expression of LDH encoding genes in *Dv*H cells. The expression level is indicated by the percentage relative to the copy number of *DVU3027* (∼60,000 copies, 100%). The copy number of the transcripts was determined from absolute quantification by using genomic dilutions as standard and expressed per μg of total RNAs. **(B)** Expression of LDH-encoding genes in Δ26-28 (black stick) and Δ32-33 (white stick) mutants compared with that of the *Dv*H WT strain. Data were obtained from biological and technical duplicates. For significant expression changes, a cutoff fold change of twofold was considered.

**FIGURE 7:**
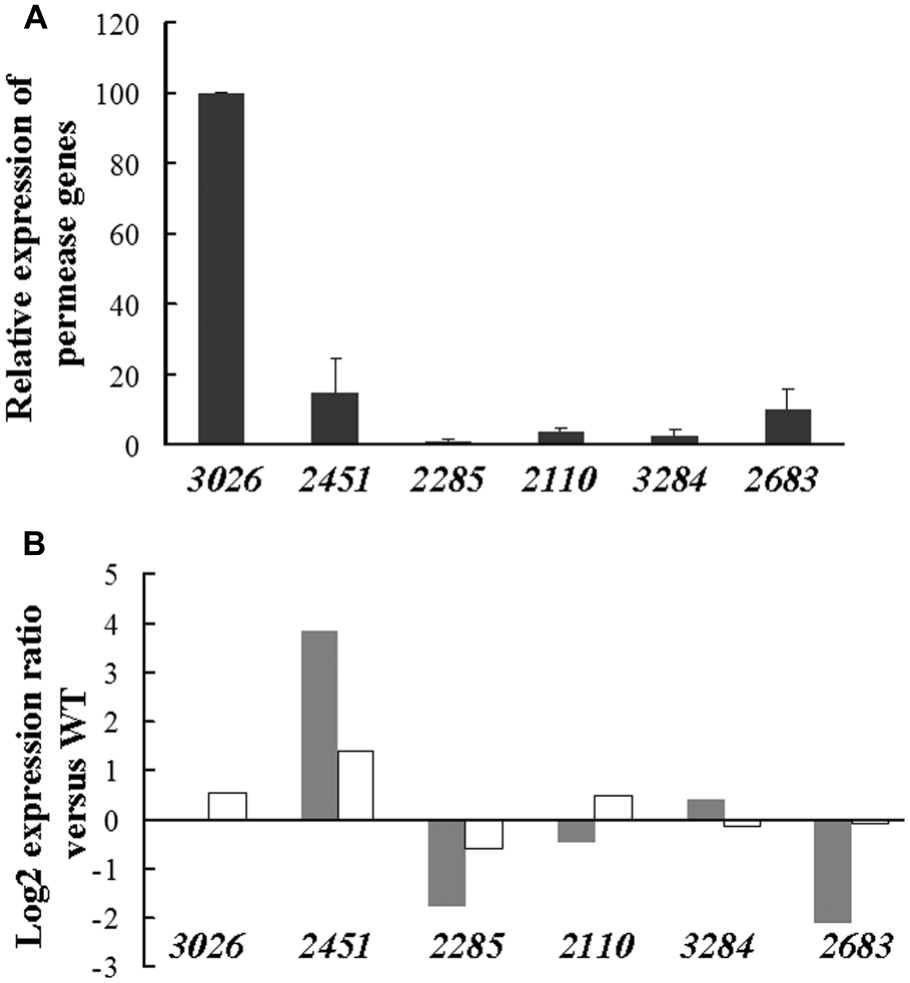
**Transcriptional analyses of predicted lactate permease-encoding genes. (A)** Relative expression of lactate permease-encoding genes in *Dv*H cells. The expression level is indicated by the percentage relative to the copy number of *DVU3026* (∼170,000 copies, 100%). The copy number of the transcripts was determined from absolute quantification by using genomic dilutions as standard and expressed per μg of total RNAs. **(B)** Expression of lactate permease-encoding genes in Δ26-28 (gray stick) and Δ32-33 (white stick) mutants compared with that of the *Dv*H WT strain. Data were obtained from biological and technical duplicates. For significant expression changes, a cutoff fold change of twofold was considered.

To obtain information on the functional role of LDH orthologs, expression of the corresponding encoding genes was measured in *Dv*H WT cells grown on D-lactate + L-lactate medium. The data represented in **Figure 6A** allowed for the classification of these genes into two groups according to their expression level. The first group included the genes *DVU0253, DVU1783*, and *DVU2784*, which exhibited a high expression level under these conditions similar to that of the genes belonging to the operon luo. The second group, which included all of the remaining genes, displayed a much lower expression level (∼5–10% of the previous level). An analysis of the expression of these genes in the Δ26-28 mutant showed that *DVU3071, DVU0253*, and *DVU0390* were down-regulated (8.8-fold, 3.7-fold, and 4-fold, respectively) compared with the WT cells, whereas *DVU2784* was up-regulated (3.7-fold) in this mutant (**Figure 6B**). The other LDH-encoding genes did not exhibit significant expression changes, and the expression of the LDH genes were not altered by the deletion of the *DVU3032-33* genes, with the exception of *DVU2784*. This latter gene was almost sixfold repressed, and it displayed inverse regulation in the two mutants. For the transport of lactate, six genes were putative lactate permease genes in the *Dv*H genome, but only *DVU3026* was highly expressed (**Figure 7A**). In the Δ32-33 mutant, no variation in the transcription level were observed for any of the genes; however, the expression of *DVU2285* and *DVU2683* was altered in the Δ26-28 mutant, with ∼fourfold and ∼fivefold repression, respectively (**Figure 7B**). In this mutant, which did not include *DVU3026, DVU2451* expression was increased by ∼ 14 fold, corresponding to the highest change. Note that the DVU2451 protein shared 88% sequence identity and 95% sequence similarity to DVU3026.

### Proteins Encoded by the Operon Luo Co-Migrate in Non-Denaturing Gel

**FIGURE 8:**
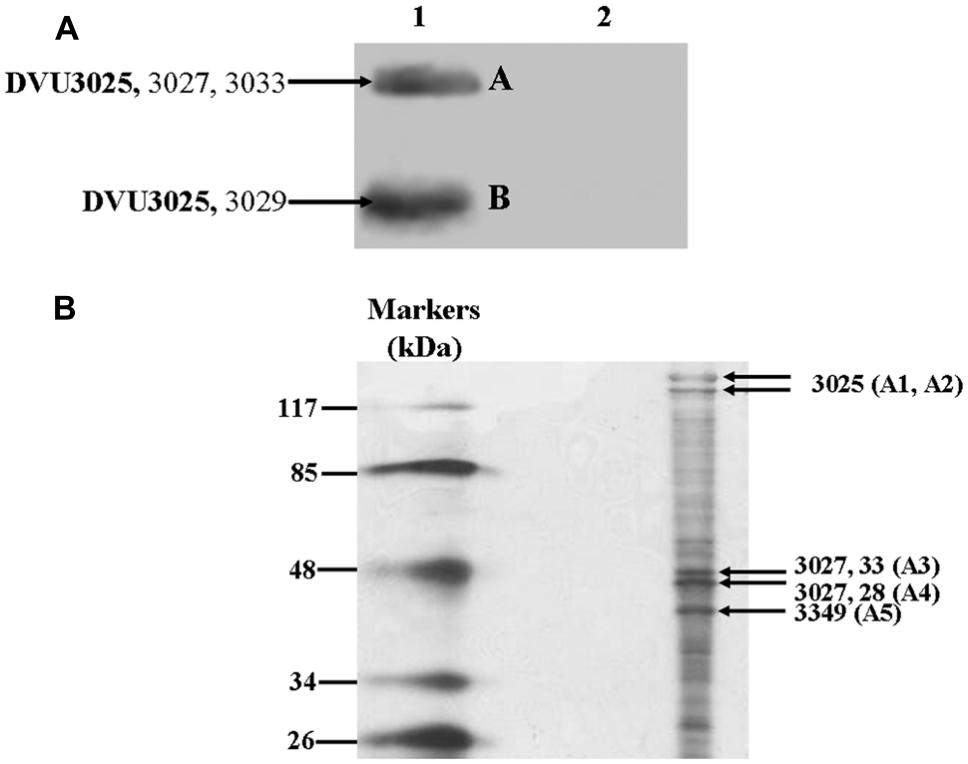
**In-gel PFOR activity and protein identification. (A)** Separation of *Dv*H protein crude extract on non-denaturing 7% PAGE and PFOR activity staining in the presence of pyruvate and coenzyme A (lane 1) and in their absence (lane 2). The proteins identified by LC–MS/MS in activity bands A and B are indicated. **(B)** Protein profile of the PFOR activity band A after PAGE on 12% denaturing gel. Proteins identified by LC–MS/MS are indicated and reported in Supplementary Table S4.

The free diffusion of enzymes in the cytoplasm together with passive diffusion of the substrates/products is commonly accepted; however, the presence of superstructures is a tempting idea as the assembly of several enzymes in a supercomplex should improve the efficiency of the corresponding metabolic network. Interestingly, an analysis of the LDH activity band (**Figure 4**) led to the identification of three proteins, DVU3025 (or PFOR), DVU3026 (or lactate permease), and DVU3029 (or phosphate acetyl transferase), in addition to the LDH subunits (Supplementary Table S3). To support these data, cell extracts of *Dv*H were prepared, separated by non-denaturing gel electrophoresis, and assayed for PFOR activity because PFOR is central to the lactate pathway. Two bands were stained with methyl viologen in the presence of pyruvate and CoA, whereas PFOR activity band was not detected when the two substrates were omitted (**Figure 8A**). The two protein bands were excised and identified by LC–MS/MS, with DVU3025 observed in the two bands (Supplementary Table S4). These results confirmed that the DVU3025 protein was the main source of PFOR activity in *Dv*H cells. In addition, two LDH subunits were also identified in band A, whereas Pta (DVU3029) was identified in band B. The PFOR activity bands were then excised and electrophoresed on SDS-PAGE. Several intense protein bands were detected from electrophoresis of band A (**Figure 8B**). The most intense protein bands were excised and analyzed by MS, which identified three LDH subunits (DVU3033, DVU3027, DVU3028) in addition to DVU3025 (Supplementary Table S4). We also identified DVU3349 from electrophoresis of band A. This protein is annotated as a pyruvate flavodoxin/ferredoxin oxidoreductase, thiamine diP-binding domain protein and could be one of the four subunits of a heterotetrameric PFOR as described in *Pyrococcus furiosus* (Kletzin and Adams, 1996). The same protocol performed on band B (**Figure 8A**) allowed us to detect only a few faint protein bands, and only DVU3025 was identified (data not shown). These data showed that PFOR, lactate permease, Pta, and LDHs co-migrated in non-denaturing gel. All together, the data described above suggest that the operon luo encodes a functional cytoplasmic supermolecular structure aimed at oxidizing lactate and producing ATP.

## Discussion

Our interest in one of key enzymes for anaerobic metabolism, pyruvate-ferredoxin oxidoreductase, led us to focus on its physiological role in *Desulfovibrio*. All attempts to date to delete the encoding gene (*por*) in *Dv*H have been unsuccessful despite the use of sources of electron donors other than lactate, such as hydrogen or formate (unpublished results). It is noteworthy that transposon mutations were not found in the *por* gene (mutant collection of Prof. J. Wall, described on the Website^2^). Overall, these findings suggest that this gene is essential. Consequently, PFOR would be the major enzyme that oxidizes pyruvate despite the presence of multiple genes encoding a number of alternative oxo-organic acid oxidoreductases that could react with pyruvate (Heidelberg et al., 2004). The *Dv*H genome analysis showed that the *por* gene is surrounded by closely related genes that could be involved in the PFOR function. Among these genes, those that encode AckA and Pta were present in only one copy per genome of *Dv*H (Heidelberg et al., 2004), and as with the *por* gene, transposon mutations were not obtained. To decipher the relationships between these genes, we first assessed the operon structure of the genome region and confirmed that the *por* gene is the first gene of a nonacistronic unit (*DVU3025 to DVU3033*) that we referred to as the operon luo, and we mapped the 5′ start of the transcript at nt 3143640. This operon structure is consistent with data obtained from high-throughput analyses in which the same transcript start was determined by 5′RNA-sequencing experiments (Price et al., 2011). The prediction of two distinct σ-dependent promoters (σ70 and σ54) in the region upstream of the transcription start site indicated that the operon expression was highly regulated. Systematic mapping in *Dv*H of the two component RRs of gene targets revealed that the operon luo was regulated by at least four regulators: DVU3023, DVU0539, DVU0621, and DVU1083. Except for DVU1083, these RRs belong to the NtrC family of σ54-dependent RRs (Rajeev et al., 2011). DVU1083 is an ortholog of the *E. coli* PhoB, an RR that activates transcription by interacting with the σ70 subunit (Blanco et al., 2011) and could be related to the presence of a -10 element upstream the transcript start of the operon luo (**Figure 3**). To stop transcription, an intrinsic rho-independent terminator was detected several nucleotides downstream of the stop codon of *DVU3033* by tiling data (Price et al., 2011). This typical terminator allowed for an efficient transcription stop of the operon luo, whereas a number of transcripts appeared to have unspecific 3′ ends in *Dv*H (Price et al., 2011). Because all of the transcription steps were tightly controlled, it is likely that the operon luo is an important pathway for *Dv*H metabolism.

An analysis of the genes surrounding the *por* gene showed that they are involved in the lactate oxidation pathway. Indeed, sequence analyses used in conjunction with genetic techniques and activity measurements were capable of identifying DVU3027-28 and DVU3032-33 as two heterodimeric LDHs that catalyze the oxidation of the D-lactate and L-lactate stereoisomers into pyruvate, respectively. These novel enzymes are homologs to the machinery for lactate utilization, which was first described in *Shewanella* (Pinchuk et al., 2009) with a non-identical multisubunit composition compared with the monomeric or tripartite *Shewanella* LDHs (Pinchuk et al., 2009). Consequently, we propose renaming the *DVU3027* and *DVU3028* genes to *dld-IIA* and *dld-IIB* genes and the *DVU3032* and *DVU3033* genes to *lldG* and *lldH* genes (**Table 1**). The observation that deletion of these genes does not induce a lethal phenotype on lactate-sulfate medium must be correlated with a high redundancy of lactate utilization machinery in *Dv*H (Keller and Wall, 2011). An expression-level analysis of all of the predicted LDH genes identified the best candidates for complementing the LDHs of the operon, particularly *DVU0253* and *DVU1781-83*, the closest homologs of the essential *Shewanella* LDH genes, which were the most expressed. However, we observed an intriguing result in the Δ26-28 mutant in which three predicted D-LDH genes, including *DVU0253*, were repressed. This observation could explain why this mutant was more affected overall than the Δ32-33 mutant. An exact interpretation of the observed functional redundancy would require a fastidious case-by-case investigation. The interpretation of lactate import into the cell was easier because the only highly expressed gene was the operon luo *DVU3026* gene, suggesting a primary role for lactate utilization. The importance of *DVU3026* was strengthened by the high transcriptional induction of *DVU2451* in the mutant Δ26-28 (**Figure 7**). Therefore, we can postulate that DVU2451 can functionally compensate for the absence of DVU3026 because they share 88% sequence identity. We propose that DVU3026 is the main transportation permease for both isomeric forms of lactate as all so far characterized lactate permeases (e.g., LldP of *E. coli*; Núñez et al., 2002). As for the operon luo, the lactate permease gene *DVU2451* is targeted by various response regulators. Of the three RRs identified, two are shared with the operon (DVU3023 and DVU0539; Rajeev et al., 2011). Rajeev et al. (2011) proposed that these RRs and their targets constituted a highly interconnected and feedback-controlled regulatory module for controlling lactate utilization. This finding was confirmed by our results, which indicated that the absence of DVU3026 induced the expression of the parolog DVU2451.

**FIGURE 9:**
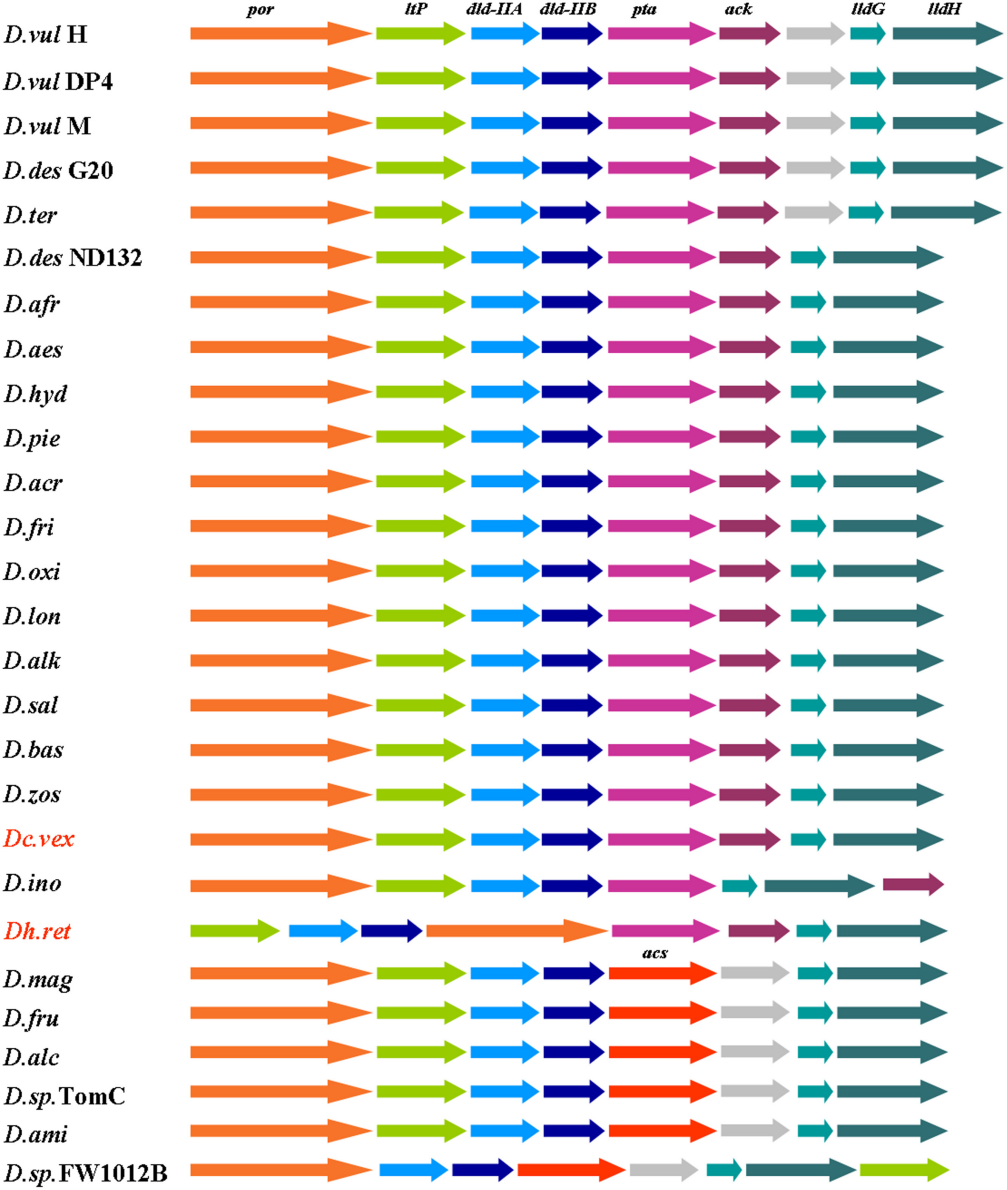
**Conservation of the organization of the operon luo in SRM.** *D.vul* H: *D. vulgaris* Hildenborough; *D.vul* DP4: *D. vulgaris* DP4; *D.vul* M: *D. vulgaris* Miyazaki; *D.des* G20: *D. desulfuricans subsp desulfuricans* G20; *D.ter*: *D. termitidis*; *D.des* ND132; *D. desulfuricans* ND132; *D.afr*: *D. africanus* Walvis Bay; *D.aes*: *D. aespoeensis*; *D.hyd*: *D. hydrothermalis* sp. AM13; *D.pie*: *D. piezophilus nov* C1TLV30; *D.acr*: *D. acrylicus*; *D.fri*: *D. frigus*; *D.oxi*: D. *oxiclinae*; *D.lon*: *D. longus*; *D.alk*: *D. alkalitolerans*; *D.sal*: *D. salexigens* DSM 2638; *D.bas*: *D. bastinii*; *D.zos*: *D. zostrae*; *D.ino*: *D. inopinatus*; *D.mag*: *D. magneticus*; *D.fru*: *D. fructosivorans*; *D.alc*: *D. alcoholivorans*; *D.sp.* TomC: *D. sp. TomC*; *D.amin*: *D. aminophilus*; *Dc.vex*: *Desulfocurvus vexinensis*; *Dh.ret*: *Desulfohalobium retbaesense*. *por, ltP, dld-IIA, dld-IIB, pta, ack, lldG, lldH*, and *acs* encode for PFOR, lactate permease, D-LDH, Pta, Ack, L-LDH, and acetyl-CoA synthetase, respectively.

Altogether, our results revealed that the proteins encoded by the operon luo constitute the major lactate oxidation pathway in *Dv*H, and these results are consistent with the recent fitness data on *D. alaskensis* mutants. Based on these data, the major LDHs in this bacterium appear to be Dde_3239:Dde_3240 and Dde_3244:Dde_3245, which are homologs of the *Dv*H operon luo LDHs (Price et al., 2014). Moreover, good conservation of the operon luo was observed in *Desulfovibrio* species. Of the 31 genomes analyzed, 19 genomes contained all of the genes described in this study. Usually, the *DVU3031* homolog gene, which is not involved in the lactate oxidation pathway, was absent (**Figure 9**). In addition, the genes encoding Ack and Pta in six other species were replaced by one gene encoding a putative ADP-forming acetyl-CoA synthetase that catalyzes acetate formation and synthesizes ATP from acetyl-CoA in various protists and *Archaea* (Bräsen et al., 2008; Tielens et al., 2010), (**Figure 9**). Interestingly, the operon luo is also conserved in other genera of sulfate-reducing bacteria, such as *Desulfohalobium* and *Desulfocurvus* (**Figure 9**). These data further substantiate the predominant role of the lactate oxidation pathway encoded by the operon luo, and they suggest that the coordinated transcription could improve the efficiency of this metabolic pathway. Identification of several operon enzymes in the LDH and PFOR activity bands suggests that all proteins would interact together in a lactate-oxidizing supermolecular structure associated to the membrane and facing the cytoplasmic compartment. The localization of this structure is strengthened by previous data showing that LDH activity was associated to the membrane in *Desulfovibrio* and *Campylobacter* cells (Stams and Hansen, 1982; Thomas et al., 2011). Further studies should be performed to address the occurrence of this supra-molecular complex that oxidizes lactate and produces ATP by substrate level phosphorylation in sulfate-reducing bacteria.

## Conflict of Interest Statement

The authors declare that the research was conducted in the absence of any commercial or financial relationships that could be construed as a potential conflict of interest.

## Footnotes

1 http://www.microbesonline.org/

2 http://desulfovibriomaps.biochem.missouri.edu/mutants/tn/DvH-Tn_lib.html

## Abbreviations

Ack: acetate kinase
CoA: coenzyme A
*D.*: *Desulfovibrio*
*Dv*H: *Desulfovibrio vulgaris* Hildenborough
LDH: lactate dehydrogenase
PFOR: pyruvate-ferredoxin oxidoreductase
Pta: phosphate acetyltransferase
*S.*: *Shewanella*
SRM: sulfate-reducing microorganisms
RR: response regulator
