# Supplementary material for: The primary pathway for lactate oxidation in Desulfovibrio vulgaris
Source: Front Microbiol. 2015 Jun 26;6:606. doi: 10.3389/fmicb.2015.00606 (PMC4481167; doi:10.3389/fmicb.2015.00606)
Supplement: Supplementary file 1 [file Data_Sheet_1.DOC]

**Supplementary Material**

**The primary pathway for lactate oxidation in *Desulfovibrio vulgaris***

**N. Vita^1, #, §^, O. Valette^1, §^, G. Brasseur^1^, S. Lignon^2^, Y. Denis^3^, M. Ansaldi^1^, A. Dolla^1^, and L. Pieulle^1^,** ^*^

^1.^From the Aix-Marseille Université, CNRS, LCB-UMR7283, Marseille, France

^2.^Plate-forme Protéomique (IBISA Marseille-Protéomique), IMM-FR3479, Marseille, France

^3.^Plate-forme Transcriptomique, IMM-FR3479, Marseille, France

**^§^**These two authors contributed equally to this work

**^#^**Present address: N. Vita, Institute for Cell and Molecular Biosciences, Cookson Building, University of Newcastle upon Tyne, Newcastle upon Tyne, NE2 4HH, United Kingdom

Running title: *Lactate oxidation in Desulfovibrio*

^*^Correspondence: L. Pieulle, Institut de Microbiologie de la Méditerranée, Laboratoire de Chimie Bactérienne, 31, chemin Joseph Aiguier, 13402 Marseille, Cedex 20, France. [pieulle@imm.cnrs.fr](mailto:pieulle@ibsm.cnrs-mrs.fr)

**Keywords:** sulfate-reducing bacteria, *Desulfovibrio*, anaerobic lactate oxidation, lactate dehydrogenase, pyruvate-ferredoxin oxidoreductase

**TABLE S1: Primers DNA sequences and plasmids used in this study**

**TABLE S2: Primers DNA sequences used for qRT-PCR**

**TABLE S3: Proteins identification by mass spectrometry from the LDH activity band**

**TABLE S4: Proteins identification by LC-MS/MS from the PFOR activity bands**

**FIGURE S1. Sequences alignment of DVU3027 and DVU3028 with *S. oneidensis* D-lactate dehydrogenase.**

**FIGURE S2. Sequences alignment of DVU3032 and DVU3033 with *S. oneidensis* L-lactate dehydrogenase.**

**TABLE S1: Primers DNA sequences and plasmids used in this study**

| **Primers** | **5’ to 3’ Sequence** |
| --- | --- |
| *For transcriptional analyses* |  |
| 1 | CGGAATTCTTGAAGGGGTGCTCTACGCCATGC |
| 2 | TTGGTACCTCTGACCGAAGATGTTCTTCTTGCC |
| 3 | TGAGAACTGCACCGTCTCTG |
| 4 | TGAAGACAAGGCAGATGACG |
| 5 | CCCTTCATGCTGTACGGTCT |
| 6 | AGGCTGTTGGTGAGGATGAC |
| 7 | CCAAGTGGATGGAGAAGGAG |
| 8 | CACAGGAGGCACTTGTTCAG |
| 9 | ACGACCTGTCGAAGCAGATT |
| 10 | ATTGATGATGGGCCTGAAGA |
| 11 | ACACCGGCAACAACACCTAC |
| 12 | TCGAAGGTCAGCTTCTCCTC |
| 13 | GTGCCTACTACGCCGTTCTC |
| 14 | CACCTCCTGGACGAGCAT |
| 15 | CCCGATGAACTCATACGACA |
| 16 | TTCTCTTCACAGCCGGAGAT |
| 17 | AGCTCCAACAGCGAAGAACT |
| 18 | TCCTTGCGGTATTCCTTCAG |
| 19 | ATCGACTTCAGCTCGTTCGT |
| 20 | GTGCCCCTGTAGACGAAGAA |
| *For identification of the transcript start site* |  |
| DVU3025rev | TCTCCTTTTTCTGCCCTGTG |
| DVU3025fwd | CCATCCACGAGAAACGACA |
| *For construction of deletion mutant* |  |
| cat-f | CTAGTCTAGAGGCGCGCCACTAGTTCTCACTTCCCTGTTAAG |
| cat-r | ATCCAAGCTTAGATCTCAATTGCGAATTTCTGCCATTCATCCGC |
| A3031-f | ATggcgcgccACCATGCAGGTCGAGAACTT |
| A3032-r | GCactagtAACTGGCAGGCTTCCTTCTT |
| B3033-f | CGCcaattgAAACTCGACAACGTCGAAGC |
| B3035-r | ATGATaagcttTGTTCGAGCATGACCTTCTG |
| **Plasmids** |  |
| pNOT19 | Cloning vector pUC19, *Nde*I site replaced with *Not*I site (Schweizer, 1992) |
| pUC19Cm | pUC19 containing the *cat* gene (Fu and Voordouw, 1997) |
| pNOTCmΔ | This study |

**TABLE S2: Primers DNA sequences used for qRT-PCR**

| **Name** | **5’ to 3’ Sequence** |
| --- | --- |
| **For LDH genes:** |  |
| Q3027L | TCATCGAGATCAACGAGCAG |
| Q3027R | AAGAACTCGATGCCCATGAC |
| Q3028L | GCGACAGGGTCTCGTATGAT |
| Q3028R | TCCAGTTCCTCGATCTGCTT |
| Q3033L | GCCATCAAGTCCAAGTCGAT |
| Q3033R | CTTGGTCACTTCGCTGAACA |
| Q0826R | TCCTTGTCGTAGTGCGAGAC |
| Q0826L | CTGTGGCATCAGTGGGTATG |
| Q0827R | GAAAGCTGACGGTGCACATA |
| Q0827L | GCAACATCCACGTGAACATC |
| Q0390L | CGTATCGACGACGAGACTCA |
| Q0390R | GCATGAGCGCCTGTATCTG |
| Q0253L | CTTGTCATCTTCCCGACCAT |
| Q0253R | CTGCTCTTCGAGCTTCGACT |
| Q3071L | ACAAGGGTTCCATCTTCGTG |
| Q3071R | GGTCGAGGTTCTTCGTGAAA |
| Q1783L | AGAGTTGTTGTGGGCAACCT |
| Q1783R | GAGTTCGAAGACCCGTTCAC |
| Q1412L | GGCTACGACAAGGTGGATGT |
| Q1412R | GCGTAGAGTCCCAGAAGCAC |
| Q2784L | TCATCCATGAGAGCGGTTTT |
| Q2784R | CAAGCTCTTCAGCCGTCTTC |
| Q0600L | ATCCTGCATGATGAGCACAG |
| Q0600R | CTATAACGCGGCAAGATGCT |
| **For lactate permease genes:** |  |
| Q3026L | CCTTGTCTTCAACTGGTTCT |
| Q3026R | AGGATGACGATCATGAAACC |
| Q2451L | CAGAATGTGAGCCGAGACAA |
| Q2451R | AGAGACCGGAAAGCTGTTGA |
| Q2110L | AGCGGTACGCAGTTCATCTT |
| Q2110R | GAGACCCCAGAAGAACACGA |
| Q3284L | TGGTGTTCTTCTGGGGTCTC |
| Q3284R | GCAGACCGGAGAGAAGAATG |
| Q2285L | TCATCTTCTCGCTCATCGTG |
| Q2285R | ATGCTGCGCCAGATTATAGG |
| Q2683L | ATCTTCCTGCTGTTGCTCGT |
| Q2683R | GTCTCCTGCATCACGGAAAT |
| **For *por* operon genes:** |  |
| Q3025L | CGTGTACGACAACATGAAGGTC |
| Q3025R | TTCTTGGAGTCGTAGGCAAAGT |
| Q3029L | TGAACGACCCCACCTACTTC |
| Q3029R | CTTGAGGCACATCAGGAACA |
| Q3030L | ACACGCTGATGAACAAGCAG |
| Q3030R | GACCGAGAACGGCGTAGTAG |
| **For 16S ribosomal RNA gene:** |  |
| QrrsAL | TGGGGAGCAAACAGGATTAG |
| QrrsAR | CACATACTCCACCGCTTGTG |

**TABLE S3: Proteins identification by mass spectrometry from the LDH activity band**

| **Mascot Score** | **% sequence coverage** | **Number of unique identified peptides** | **Genome annotation** | **Locus Tag** | **MW (Da)** |
| --- | --- | --- | --- | --- | --- |
| 1 418 | 23 | 31 | PFOR | DVU3025 | 131,462 |
| 214 | 7 | 5 | L-lactate permease | DVU3026 | 60,813 |
| 354 | 23 | 7 | Glycolate oxidase, subunit GlcD | DVU3027 | 49,347 |
| 370 | 27 | 11 | Iron-sulfur cluster-binding protein | DVU3028 | 45,924 |
| 183 | 6 | 4 | Phosphate acetyltransferase | DVU3029 | 76,782 |
| 358 | 11 | 7 | Iron-sulfur cluster-binding protein | DVU3033 | 79,655 |

**TABLE S4: Proteins identification by LC-MS/MS from the PFOR activity bands**

| **Band**  **name^a^** | **Sequest score** | **% sequence coverage** | **Number of identified peptides^b^** | **P (pro)^c^** | **Genome annotation** | **Locus Tag** | **MW (Da)** |
| --- | --- | --- | --- | --- | --- | --- | --- |
| A | 250.19 | 28.31 | 32 | 3.25 E-07 | PFOR | DVU3025 | 131,462 |
| A | 40.13 | 10.85 | 4 | 1 | Glycolate oxidase, subunit GlcD | DVU3027 | 49,347 |
| A | 20.16 | 3.35 | 3 | 1 | Iron-sulfur cluster binding protein | DVU3033 | 79,655 |
| B | 160.19 | 15.47 | 18 | 1.87 E-07 | PFOR | DVU3025 | 131,462 |
| B | 20.18 | 4.26 | 2 | 1 | Phosphate acetyltransferase | DVU3029 | 76,782 |
| A1 | 140.18 | 13.66 | 23 | 2.12 E-07 | PFOR | DVU3025 | 131,462 |
| A2 | 438.15 | 36.3 | 27 | 1.28 E-08 | PFOR | DVU3025 | 131,462 |
| A3 | 50.20 | 8.93 | 7 | 8.86 E-07 | Iron-sulfur cluster binding protein | DVU3033 | 79,655 |
| A3 | 20.12 | 2.60 | 3 | 1 | Glycolate oxidase, subunit GlcD | DVU3027 | 49,347 |
| A4 | 30.14 | 1.89 | 3 | 4.10 E-05 | Glycolate oxidase, subunit GlcD | DVU3027 | 49,347 |
| A4 | 30.15 | 5.00 | 5 | 5.94 E-05 | Iron-sulfur cluster binding protein | DVU3028 | 45,924 |
| A5 | 60.23 | 26.60 | 8 | 8.91 E-08 | pyruvate flavodoxin/  ferredoxin  oxidoreductase, thiamine diP-binding domain protein | DVU3349 | 38,471 |

^a^ Results obtained from PFOR activity bands revealed in native gel (A and B) and from band A separated on a SDS-PAGE (A1 to A5), (Fig. 8).

^b^ Identified proteins must have at least 2 unique peptides in first position.

^c^ When P is high, the identification was checked manually.

Fu, R., and Voordouw, G. (1997) Targeted gene-replacement mutagenesis of dcrA, encoding an oxygen sensor of the sulfate-reducing bacterium *Desulfovibrio vulgaris* Hildenborough. *Microbiology* 143 (Pt 6), 1815-1826.

Schweizer, H. P. (1992) Allelic exchange in *Pseudomonas aeruginosa* using novel ColE1-type vectors and a family of cassettes containing a portable oriT and the counter-selectable *Bacillus subtilis* sacB marker. *Mol. Microbiol.* 6, 1195-1204.

Dld-II -------------------MTDDPVRRFAWSTDASYFRIVPEVVVHAETLEQVKLTLTVA

DVU3027-28 MPSASLIKEFEAIIGKENVFTSEPDRQSYAYDSAVLDQVVPALVLRPTETEQLGKLVKLC

:*.:* *: .* ::** :*::. **: :.:.

Dld-II RKHNAPVTFRAAGTSLSGQAIGEG--ILLILGHDGFRKIEVSSDAKQITLGAAVIGSDAN

DVU3027-28 YENDHPITVRGAGTNLSGGTIPDKREGIVILTNSLNKIIEINEQDLYAVVEPGVVTAKFA

::: *:*.*.***.*** :* : ::** :. : **:..: .: ..*: :.

Dld-II AVLAPLNRKIGPDPATIASAKIGGIVANNASGMCCGTAQNSYQTIASAKLLFADGTELDT

DVU3027-28 AEVAKRGLFYPPDPGSQAVSTLGGNVAENAGGLRGLKYGVTKDYVMGIEFFDVNGGLVKT

* :* . ***.: * :.:** **:**.*: . : : : . ::: .:* :.*

Dld-II GCEKSKAEFAKTHGKLLQDLSELSHLTRHNSALAERIRKKYSIKNTTGYGINSLIDFTDP

DVU3027-28 GS--------------------------------------RTVKCVTGY-----------

*. ::* .***

Dld-II FDIINHLMVGMEGTLAFINEVTYHTVNEAKFKASAMAVFHNMEDAARAIPLINGESVS--

DVU3027-28 --NLAGLMAASEGTLGVFSQITLKLVPPPKASKAMMAVFDDVNKASEAVAAIIAAHVVPC

: **.. ****..:.::* : * .* . : ****.:::.*:.*:. * . *

Dld-II AAELLDWPSIKAVTGKPGMPDWLSELPALSAILLIESRADDAQTLEHYTQDVTAKLAGFD

DVU3027-28 TLEFMDKSSINYVEDFTKAG-----LPREAAAILLIEVDGHPAQVEDDAATVVKALN-AS

: *::* .**: * . . ** :* :*: . ... :*. : *. * .

Dld-II FIRPMEFSTNPAVYDKYWAMRKGLFPIVGGERPKGTSVIIEDVAFELEHLAAAAHDITEL

DVU3027-28 GATEVHVAKDAAEKFKLWEARRNALPALARAR---ATTVLEDATVPRSQIPAMVKAINDI

:..:.:.* * * *:. :* :. * ::.::**.:. .::.* .: *.::

Dld-II FHKHGYPEGCIYGHALAGNFHFIITPAFTTQADIDRFHAFMDDIADMVINKYNGSMKAEH

DVU3027-28 AKKHNIAIG-TFGHAGDGNLHPTILCDRRDKHEFERVESAVDEIFDVALS-LHGTLSGEH

:**. . * :*** **:* * : :::*..: :*:* *:.:. :*::..**

Dld-II GTGRAVAPFVEKEWGQDAYTLMKNIKQVFDPQGILNPGVILNDDSNIHVKNIKPCPVVDD

DVU3027-28 GIGLAKSKWMEKETSKATIEYSRNMKRAIDPKYILNPGKIIG-AMADLTKLAKMLQELDD

* * * : ::*** .: : :*:*:.:**: ***** *:. .* * :**

Dld-II FVDKCIECGFCEKTCPTSALNFSPRQRIATLREIERLEQSGDKAAAAKMRADAKYDVIDT

DVU3027-28 HMVACMKCGMCQAVCPVFAETMKEAD--VTRGKIALLENLAKEMVSDPEGVQEKLNKCLL

.: *::**:*: .**. * .:. : .* :* **: ..: .: .: * :

Dld-II CAACQLCTIACPVDNSMGQLVRKLRTPYISTTEQKVLDFQAKHFG--AVNQVISTGFDVL

DVU3027-28 CGSCGANCPSGVKIMDIFLRARCIVNSYMGLSPVKKAILRGMLTNPKLFNALLDMGSVFQ

*.:* : .: .* : ..*:. : * ::. . .* ::. * .

Dld-II GVIHKITGDGITNALMKTGRLISKEVPYWNPDFPKGGKLPK--PSPAKAGQETVVYFPAC

DVU3027-28 GLFTTKVNDLLGSSCSKILSPIIGDRHFVGLASKSLHSRIKSLDTPAGKSGVKVAFFPGC

*:: . ..* : .: * * : : . . . * :** . .*.:**.*

Dld-II GGRTFGPTPKDPDNRTLPEVVVTLLERAGYNVITPEKTRDLCCGQMWESKGDFKNADAKR

DVU3027-28 LG--------DKMFTSVADACLKVFSHHGVGVYMPEG--MACCGIPSLASGDRVSYDKLV

* * ::.:. :.::.: * .* ** *** :.** . *

Dld-II QELIDVLSKMSNGGKIP---VLVDALSCTYRTLTGNPQVQITDLVEFMHDKLLDKLSINK

DVU3027-28 KLNLDLFAKGKFDYLVTPCATCTATIKEIWPKMMGDYPFEMRKQIEELEKKTMDVNAFVV

: :*:::* . . :. . . ::. : .: *: .:: . :* :..* :* ::

Dld-II KVN--------------VALHLGCSARKMK---LEPKMQAIANACSAQVLKPAGIECCGY

DVU3027-28 DVLGVTPAADAPKGNVKVTFHDSCHMKKSLGVTAQPRNLIRMNPKYDLVEMAECDRCCGS

.* *::* .* :* :*: *. * . .***

Dld-II AGEKGLYKP----EINASALRNIKKLIPVEVKEGYYANRMCEVGLTQHSG--ISYRHLAY

DVU3027-28 GGSFNLYHYDLSKQIGERKRQNIVDSGAQVVSTGCPACMLQMTDMLSQHGDRVAVKHCIE

.*. .**: :*. :** . . *. * * : ..: .: * :: :*

Dld-II LLEECSR

DVU3027-28 IYADSLG

: :.

**FIGURE S1. Sequences alignment of DVU3027 and DVU3028 with *S. oneidensis* D-lactate dehydrogenase.** Dld-II: D-lactate dehydrogenase of *Shewanella oneidensis*. Sequences of DVU3027 and DVU3028 are highlighted in blue and green, respectively. In the N-terminal sequence of Dld-II and in DVU3027 sequence, an essential histidine is conserved in a degenerated motif GEHGD (highlighted in grey). This histidine is conserved in enzymes that bind lactate. The two canonical binding motifs for [4Fe-4S] clusters are highlighted in pink. Cysteine-rich sequence motifs (CX_n_CCGX_m_CXXC), designated CCG motif are highlighted in yellow. Note that for the two copies of the CCG motif in Dld-II the last conserved cysteine of the motif is replaced by another amino acid residue.

L-LDH MSSKHEILNALKLSALTNHPMPSIDVAPRVEDLVGQFETNLKTVAGTLHREGGLAALQAK

DVU3032-3033 -MSGNQDLARLMREKAEAIAATVVELKDINEAFAYALDICEKKEACQLLISGCEEKLSDN

* :: * * . . . ::: * :. :: *. * * .* *. :

L-LDH VDELIAQGLQVISLVEGVTAN---------------RDVPPTAHELRDIDYAVIPGDVGV

DVU3032-3033 AEALCEMKQKKIIAAPNLSDKEYDAFAKLCEERGIACVREGLRKHLAGIDIGFTHVTMGI

.: * : * . .:: : :.* .** .. :*:

L-LDH AENGAIWVNNKNLGHRVTPFICENLILALPIHKIVPNMHQAAKEVTLDAG----EFGVFI

DVU3032-3033 AETGTCVVSSNSEELRLASMISEFHVAVLPKSKIVATSYDAEATLNELMGTGKPHYTAFI

**.*: *..:. *::.:*.* : .** ***.. ::* :. * .: .**

L-LDH AGPSKTADIEQALVVGAHGACSLNVYLVMAYQHNHEAMGSQVHAYKADIFCRDETRVDWH

DVU3032-3033 SGPSRTADIERVLSLGVHGPLELHLILVEGMQNSKTLKEYRKELQESLDNEFLRNAMDKF

:***:*****:.* :*.**. .*:: ** . *:.: : . :: .. :* .

L-LDH SKALWLLREKRDRAAGSLPEWEQLRQLGSEIKLHTLTNLAQYLETFEQNCLANGIKVHWA

DVU3032-3033 AVAYRASRAN----AFKDIDEKAIIAEVADAKDHAAKNMDTLYAQFKAEAEKRGVKVHLA

: * * : * . : : : :: * *: .*: *: :. .*:*** *

L-LDH KDGAEHNRIVHEILASHKVKKLVKSKSMLTEECHLNPYLEQRGIEVIDTDLGERIIQLAK

DVU3032-3033 RTAAEANEIIARIARDNNCKKAIKSKSMTAEETHLNHRLEEDNVEVIETDLGEWIIQMRH

: .** *.*: .* .:: ** :***** :** *** **: .:***:***** ***: :

L-LDH MPPSHIVVPAIHMKKEEVGDLFHDKLGTKAGESDPLYLTRAARAHLREQFLSADAAMTGV

DVU3032-3033 EGPSHMVMPAIHLSRYQVADLFSEVTKQKQ-EVDIQRLVKVARRELRTHFATADMGISGA

***:*:****:.: :*.*** : * * * *.:.** .** :* :** .::*.

L-LDH NMAIADKGAVVVCTNEGNADMGANLPKLQLHSMGIDKVVPDIDSAAVLLRTLARNATGQP

DVU3032-3033 NFAVAETGTIGLVTNEGNARLVTTLPRVHVALAGLDKLVPTLHDALRSLKVLPRNATGQA

*:*:*:.*:: : ****** : :.**:::: *:**:** :..* *:.*.******.

L-LDH VTTYSAFYRGP-----QVDG--EMHVIIVDNGRTEMMKDKILAESLKCIRCGGCLNTCPV

DVU3032-3033 ITSYVTWIGGANECEACVDGRKEMHIVFLDNGRRALAEDPLFSQVLRCVRCGACANVCPV

:*:* :: *. *** ***::::**** : :* :::: *:*:***.* *.***

L-LDH YRRSGGYSYNYTIPGPIGIAVG---ATHDNTNSIAWACTLCGSCTYVCPTKVPLDKIIHH

DVU3032-3033 YRLVGGHKMGHIYIGAIGLILTYFFHGRDKARNLVQNCINCESCKHICAGGIDLPRLIKE

** **:. .: *.**: : :*::..:. * * **.::*. : * ::*:.

L-LDH HRRLKAEAGKLPYGKNAYMPLVGKFMASTTLLNCSMGAARTALRILPGSLLKPFSGAWGK

DVU3032-3033 IRARLNEEEGMPVETTLMGKMLKNRKLFHTLLRFAKWAQKPVTGGTPYIRHLPQIFAKDH

* * :* .. :: : ***. : * :.. * * * .:

L-LDH -YRELPVAPNSSFEAWFKKHR-----SLMKIALFIPCLVNQMMPDVAIATLELLEKLGHQ

DVU3032-3033 GFKALPAIADKPFRDEWETVRPRIAKPKLRIALFSGCVQDFVYPEQMKAAVKVIASQNVD

:: **. .:..*. ::. * . ::**** *: : : *: *::::: . . :

L-LDH VILPAGQTCCGQPMTNSGCFDAARSTTLKLLNAFKGVECDAIVCPAASCLVAAKENFHEF

DVU3032-3033 IDFPMDQSCCGLPVQMMGEREATIEVARQNVMAFDAARYDYIVTLCASCASHLKETYPKL

: :* .*:*** *: * :*: ..: : : **.... * ** .*** **.: ::

L-LDH DN-----SPEAQAVINKLYELTEFLHDIAPIP--AFNKPFAHKISLQLSCHGIRMLSLAT

DVU3032-3033 LTGHPEMTTRVRQFSNKIIDFSSFVHDVLGMKSDAFKGGSNEKVAYHSSCHLCRGLGVVE

. :...: . **: :::.*:**: : **: .*:: : *** * *.:.

L-LDH PSEQMGPRFNKVEAVLANIAGIDIVYPDRRDECCGFGGTFAVDEGAVSAKMGKDKAQAHA

DVU3032-3033 QPR-----------NLIAASGATYCKAEEEDVCCGFGGTFSAKFPELSAELLRKKLDNVE

.. * :* .:..* ********:.. :**:: :.* :

L-LDH ATGAQYVVGFDPSCLLHLDGLIRRQQLPIEIRHIAQVLNAAL-

DVU3032-3033 ATGAGRLVADCPGCIMQLRGGMEKRGGKVKVGHVAELLAENLK

**** :*. *.*:::* * :.:: ::: *:*::* *

**FIGURE S2. Sequences alignment of DVU3032 and DVU3033 with *S. oneidensis* L-lactate dehydrogenase.** L-LDH: L-lactate dehydrogenase of *Shewanella oneidensis*. L-LDH is a tripartite enzyme of three subunits, LldG (in grey), LldF (in white) and LldE (in yellow). Sequences of DVU3032 and DVU3033 are highlighted in blue and green, respectively. In LldG and DVU3032 sequences, an essential histidine is conserved in a degenerated motif GEHGD (highlighted in red). This histidine is conserved in enzymes that bind lactate. The two canonical binding motifs for [4Fe-4S] clusters in LldF and DVU3033 are highlighted in red. Cysteine-rich sequence motifs (CX_n_CCGX_m_CXXC), designated CCG motif are highlighted in pink. Note that for the two copies of the CCG motif in LldE the fourth conserved cysteine of the motif is replaced by another amino acid residue.
